# Ventromedial prefrontal cortex lesions disrupt learning to reward others

**DOI:** 10.1093/brain/awaf056

**Published:** 2025-02-11

**Authors:** Margot Gueguen, Jo Cutler, Daniel Drew, Matthew A J Apps, Deva Sanjeeva Jeyaretna, Masud Husain, Sanjay G Manohar, Patricia L Lockwood

**Affiliations:** Centre for Human Brain Health, School of Psychology, University of Birmingham, Edgbaston, Birmingham B15 2TT, UK; Centre for Human Brain Health, School of Psychology, University of Birmingham, Edgbaston, Birmingham B15 2TT, UK; Institute for Mental Health, School of Psychology, University of Birmingham, Edgbaston, Birmingham B15 2TT, UK; Department of Experimental Psychology, University of Oxford, Radcliffe Observatory Quarter, Oxford OX2 6GG, UK; Wellcome Centre for Integrative Neuroimaging, University of Oxford, John Radcliffe Hospital, Headington, Oxford OX3 9DU, UK; Department of Experimental Psychology, University of Oxford, Radcliffe Observatory Quarter, Oxford OX2 6GG, UK; Wellcome Centre for Integrative Neuroimaging, University of Oxford, John Radcliffe Hospital, Headington, Oxford OX3 9DU, UK; Nuffield Department of Clinical Neurosciences, University of Oxford, John Radcliffe Hospital, Oxford OX3 9DU, UK; Centre for Human Brain Health, School of Psychology, University of Birmingham, Edgbaston, Birmingham B15 2TT, UK; Institute for Mental Health, School of Psychology, University of Birmingham, Edgbaston, Birmingham B15 2TT, UK; Department of Experimental Psychology, University of Oxford, Radcliffe Observatory Quarter, Oxford OX2 6GG, UK; Wellcome Centre for Integrative Neuroimaging, University of Oxford, John Radcliffe Hospital, Headington, Oxford OX3 9DU, UK; Nuffield Department of Clinical Neurosciences, University of Oxford, John Radcliffe Hospital, Oxford OX3 9DU, UK; Department of Experimental Psychology, University of Oxford, Radcliffe Observatory Quarter, Oxford OX2 6GG, UK; Wellcome Centre for Integrative Neuroimaging, University of Oxford, John Radcliffe Hospital, Headington, Oxford OX3 9DU, UK; Nuffield Department of Clinical Neurosciences, University of Oxford, John Radcliffe Hospital, Oxford OX3 9DU, UK; Department of Neurology, John Radcliffe Hospital, Oxford OX3 9DU, UK; Department of Experimental Psychology, University of Oxford, Radcliffe Observatory Quarter, Oxford OX2 6GG, UK; Nuffield Department of Clinical Neurosciences, University of Oxford, John Radcliffe Hospital, Oxford OX3 9DU, UK; Department of Neurology, John Radcliffe Hospital, Oxford OX3 9DU, UK; Centre for Human Brain Health, School of Psychology, University of Birmingham, Edgbaston, Birmingham B15 2TT, UK; Institute for Mental Health, School of Psychology, University of Birmingham, Edgbaston, Birmingham B15 2TT, UK; Department of Experimental Psychology, University of Oxford, Radcliffe Observatory Quarter, Oxford OX2 6GG, UK; Wellcome Centre for Integrative Neuroimaging, University of Oxford, John Radcliffe Hospital, Headington, Oxford OX3 9DU, UK

**Keywords:** ventromedial prefrontal cortex, brain damage, lesion mapping, social learning, reinforcement learning, computational modelling

## Abstract

Reinforcement learning is a fundamental process through which humans and other animals attain rewards for themselves. However, to act prosocially, we must also learn how our choices reward others. The ventromedial prefrontal cortex has been independently linked to reinforcement learning and prosocial behaviour, yet its causal impact on prosocial reinforcement learning and the roles of its multiple subregions remain unknown.

Here, a large group of adults with rare focal ventromedial prefrontal cortex damage (*n* = 28) and two carefully age- and gender-matched control groups (lesions elsewhere, *n* = 21; healthy controls, *n* = 124) completed a reinforcement learning task where they learnt to win rewards for another person (prosocial), for themselves (self) or in a control condition where participants saw points, but they were not translated into rewards for either individual (no one, control condition) on separate trials. A novel computational model incorporating separate learning rates for positive and negative prediction errors best explained behaviour in all groups.

Importantly, compared to both control groups, patients with ventromedial prefrontal cortex damage were less accurate and had lower learning rates from positive prediction errors when rewarding another person relative to when no one benefitted, and higher learning rates for negative prediction errors when learning for others relative to self. Unlike controls, ventromedial prefrontal cortex lesion patients also showed a reduced self-benefitting advantage. They were equally accurate and learnt at a similar rate from positive prediction errors for self and neither individual. Strikingly, voxel-based lesion-symptom mapping revealed that damage to subgenual anterior cingulate cortex and anterior cingulate cortex gyrus specifically disrupted prosocial reinforcement learning.

These findings highlight the importance of ventromedial prefrontal cortex integrity for multiple aspects of reinforcement learning, with damage to subgenual anterior cingulate cortex and anterior cingulate cortex gyrus critical in learning to reward others.

## Teaser

Using a novel computational neurology approach in a cohort of patients with rare focal ventromedial prefrontal cortex damage, Gueguen *et al.* show that this brain region is necessary for learning to reward others, with different subregions playing distinct roles. The results have implications for the neurobiological basis of social behaviour.

## Introduction

Decades of neuroscientific research in humans and animals has argued that the ventromedial prefrontal cortex (vmPFC) is critical for learning to attain rewards for oneself. Much of this evidence comes from correlational techniques such as neuroimaging,^1-3^ which show that the vmPFC processes variables that guide learning. For example, vmPFC has been suggested to track the subjective value of rewards and signal prediction errors (PEs; representing the unexpectedness of an outcome) during rewarding outcomes.^4-6^ Relatedly, vmPFC involvement during learning has been linked to processing valence. While some have argued that vmPFC preferentially represents either rewards^7-9^ or punishments,^10,11^ meta-analyses of functional neuroimaging studies highlight that vmPFC robustly activates with both.^12,13^ A different line of research in human lesion patients also points to vmPFC involvement in probabilistic, reversal and devaluation learning.^14,15^ Indeed, while patients with vmPFC lesions can learn simple associations between a stimulus and a reward when contingencies are fixed, some evidence suggests that they may struggle to learn to update their behaviour to keep gaining rewards for themselves when contingencies are changing and uncertain.^4-6^

However, vmPFC subregions are determined based on functional descriptions rather than anatomical landmarks, and convincing evidence suggests that different portions of vmPFC may have discrete functions and connectivity.^16^ For instance, studies in non-human primates have shown that individual neurons in ventral vmPFC are preferentially activated by rewards, and neurons in dorsal vmPFC by punishment.^17^ As such, the relatively large-scale activations found in human vmPFC may result from smoothing or combining of signals from distinct sub-regions that process outcome valence or other aspects of the learning process.

Contrasting with the research suggesting its role in self-benefitting learning, evidence also highlights the crucial importance of vmPFC in learning to benefit others, termed prosocial learning.^18-20^ Several subregions of vmPFC appear specifically involved in prosocial reinforcement learning. The subgenual anterior cingulate cortex (sgACC), ventrally located within vmPFC, has been shown to track prediction errors when learning to benefit another person, but not oneself or no one.^19,20^ Dorsal to the corpus callosum, the anterior cingulate gyrus (ACCg; areas 24a and b) has been found to signal rewards for others, as well prediction errors for others’ rewarding outcomes, when matched other- and self-benefitting conditions are directly compared.^21-24^ Whether these subregions are domain-general or domain-specific for social and non-social processes is a pivotal question within social neuroscience.^21,22,25^ However, the neural recording techniques employed in this existing research cannot establish causation. Moreover, the majority of human lesion studies of vmPFC have either been case studies or tested groups of fewer than ten patients. Given typical variability in learning and prosocial behaviour, larger samples are needed to draw robust conclusions about the necessity of vmPFC for prosocial and self-benefitting reinforcement learning and the specific roles of distinct subregions. Finally, social behaviour disturbances after brain damage are some of the most common complaints from caregivers, yet the impact of focal lesions on social function is still vastly understudied compared to impacts on executive and physical functions.^26^

In this study, we aimed: (i) to test whether vmPFC is causally involved in prosocial and self-benefitting reinforcement learning; and (ii) to examine the distinct contributions of specific vmPFC subregions to prosocial and self-benefitting learning using voxel-based lesion-symptom mapping (VLSM). We tested the causal role of vmPFC in prosocial and self-benefitting reinforcement learning by comparing a large sample of patients with rare focal vmPFC lesions (*n* = 28) to two age- and gender-matched control groups: patients with lesions elsewhere (lesion controls, LC; *n* = 21), and participants with no brain damage (healthy controls, HC; *n* = 124). All participants completed a reinforcement learning task^19,27,28^ in which they had to choose between two abstract symbols probabilistically associated with rewards on each trial. A third of the trials were self-benefitting, with the points converted into bonus money for themselves. Crucially, a third of trials were prosocial, where the points earned by the participant resulted in bonus money for a real and anonymous other participant. The remaining trials displayed points, but these did not benefit anyone, creating a tightly matched control condition absent of economic reward (see the ‘Materials and methods’ section). We took a computational neurology approach, comparing multiple mathematical models to precisely quantify the participants’ learning, and using the resulting computational parameters in voxel-based lesion-symptom mapping. By integrating data-driven and theory-driven computational techniques with clinical neurology and basic neuroscience, computational neurology aims to improve our understanding of neurological dysfunction and has already allowed breakthroughs in the detection of Alzheimer’s disease.^29,30^ This approach holds great promise for advancing our understanding of the fundamental components of disorders of social behaviour.

Our findings provide converging evidence that vmPFC damage disrupts prosocial reinforcement learning, confirming the causal role of this region in learning to benefit other people. Relative to both control groups, patients with focal vmPFC damage were less accurate when learning to reward another person compared to neither individual, had aberrantly low learning rates for positive prediction errors for other compared to neither, and aberrantly high learning rates for negative prediction errors for other relative to self. Strikingly, damage to the sgACC subregion specifically disrupted prosocial learning rates from both positive and negative prediction errors, converging with previous correlational functional MRI (fMRI) results. In addition, damage to ACCg was associated with aberrant learning rates for negative prediction errors when learning to benefit another person relative to self. Finally, we also found that vmPFC damage caused a reduced self-benefitting advantage. Together, our results suggest a causal influence of vmPFC in learning to benefit other people and highlight that distinct subregions, sgACC and ACCg, play critical roles in prosocial learning. These findings could have important clinical implications for predicting social behaviour difficulties after brain damage.

## Materials and methods

### Participants

**Figure 1:**
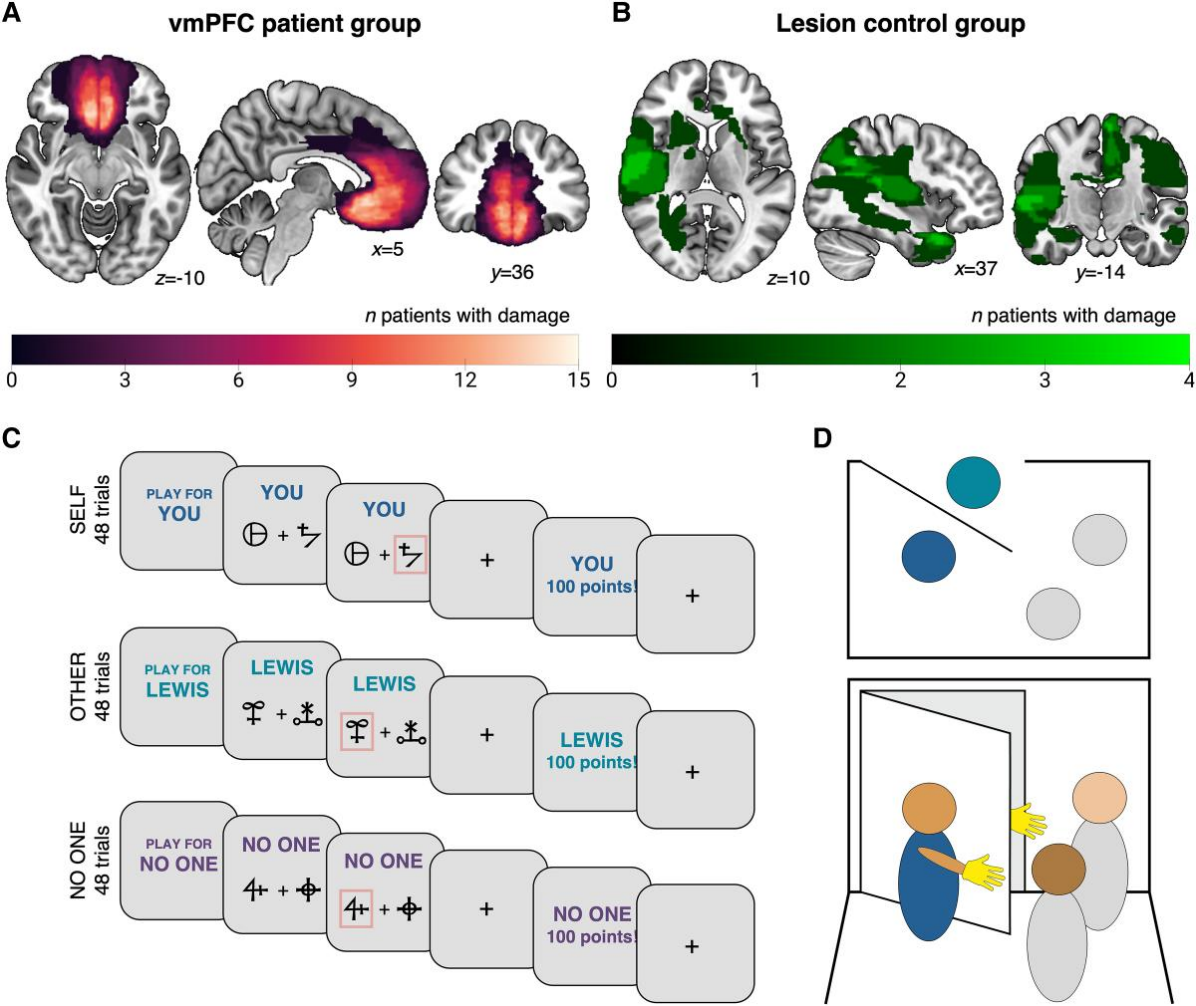
**Lesion locations and prosocial learning task with rewards for self, other or no one.** (**A**) Patients in the vmPFC group (*n* = 28) displayed focal lesions that extended across the lateral (area 13) and medial (areas 14, 11, sgACC, ACCg) regions of bilateral vmPFC. (**B**) Patients in the lesion control group (*n* = 21) also had damage primarily caused by subarachnoid haemorrhage. However, this damage did not encompass the vmPFC (see the ‘Materials and methods’ section). (**C**) Participants completed a reinforcement learning task in which they learnt the probability that two abstract symbols would lead to winning points for themselves, another individual, or neither person. One symbol was associated with a high (75%) and the other with a low (25%) probability of reward. Participants received visual feedback on whether they earned points (either 0 or 100 points) after each decision. Points were converted into money for the participant and for the other player in the self and other conditions, respectively, but points in the no one condition were only displayed and not converted into an economic reward for anyone. (**D**) The participant (dark blue) was anonymously introduced to a confederate researcher (light blue) by two experimenters (grey). *Top*: Positioning of the individuals from above. The participant and confederate researcher were on either side of a door such that they could not see each other, but they could see both experimenters. *Bottom*: The participant and confederate wore gloves and were asked to wave at each other while remaining silent. Prior to this, the experimenter informed the participant that they would be assigned to the role of ‘Player 1’ or ‘Player 2’. They were told that Player 1 would be able to earn points (translated into bonus payment) for themselves, for the anonymous confederate or for neither in the game, while Player 2 would play a different game with outcomes only affecting themselves. The participant was in fact always assigned to the role of Player 1. To eliminate the potential influences of reciprocity and reputation, the participant was informed that Player 2 was not aware they might receive additional money and that bonus payments would be made anonymously. The name used for Player 2 in the task was gender-matched to the participant. ACCg = anterior cingulate cortex gyrus; sgACC = subgenual anterior cingulate cortex; vmPFC = ventromedial prefrontal cortex.

We recruited three groups of participants: one with localised damage to vmPFC and one with lesions elsewhere from a database of 453 neurological patients, and a group of healthy controls from university databases and the community. Participants were all aged 18 or above, had normal or corrected-to-normal vision, and did not study psychology. In addition, healthy controls did not previously or currently have a neurological or psychiatric illness. One vmPFC patient was excluded due to missing data on our task. Our final sample consisted of 173 participants, including 28 patients with focal vmPFC lesions (age range: 37–76 years old, mean = 57; 15 females; Fig. 1A), 21 lesion control patients with damage elsewhere (LC; age range: 28–74 years old, mean = 56; 13 females; Fig. 1B) and 124 healthy control participants (HC; age range: 20–80 years old, mean = 53; 71 females). The three groups were matched on age (*P*s > 0.41), gender [*χ*^2^(2) = 0.34, *P* = 0.84] and did not significantly differ in terms of self-reported empathy (Questionnaire of Cognitive and Affective Empathy^31^ total score, *P*s > 0.61; affective empathy, *P*s > 0.14; cognitive empathy, *P*s > 0.62). In addition, the vmPFC group was matched to both control groups in terms of cognitive ability and visual attention (Trail Making Test^32^ Part A, *P*s > 0.40; Part B, *P*s > 0.40) and apathy (Apathy Motivation Index,^33^  *P* > 0.14). The lesion groups did not significantly differ in depression (Beck Depression Index II,^34^  *P* = 0.50).

Lesions were caused by subarachnoid haemorrhage due to aneurysm or arteriovenous malformation in 26 of the 28 vmPFC patients and in all lesion control participants. All patients had subarachnoid haemorrhage that was complicated by either vasospasm of the anterior cerebral artery or its branches, or in some cases by parenchymal haemorrhage, or in one case, as a complication after coiling, presumed embolic. Two mPFC lesions were caused by meningiomas that were resected. In all cases, the brain damage occurred at least 24 months prior to testing. Patients had no other neurological diagnoses. In terms of neurotropic medications, in the vmPFC group, one was on an anticonvulsant (levetiracetam), one was on antidepressants (amitriptyline) and one patient was on pregabalin. In the lesion controls, one was on an anticonvulsant (lamotrigine), two were on antidepressants (citalopram) and one was on pregabalin.

### Lesion identification

Of the 49 patients, 47 had MR imaging. Most patients had 1 mm isotropic T1 FSPGR MRI plus 6 mm axial T2 PROPELLER sequence. Two patients had CT imaging instead, due to metal. The source MRI images were clinical sequences optimised to assess aneurysm recurrence and hence had minimal artefact from coils or clips. A neurologist (S.G.M.) manually traced each patient's lesion onto the MNI152 template using FSL (http://fsl. fmrib.ox.ac.uk/fsl), yielding a binary mask, slice by slice, prior to behavioural testing. The mean lesion volume was 2.74 cm^3^ (SD = 2.63) and ranged from 0.02 to 9.74 cm^3^. Total lesion volume did not correlate with any of the variables included in the VLSM analysis (Supplementary Fig. 1). An overlap map was constructed for each group by counting the number of patients with damage in each voxel (Fig. 1A and B).

### Procedure

Participants took part in a single in-person testing session that began with an evaluation by a neurologist (S.G.M.). We then conducted the ‘role assignment’ procedure (see later) before the participants completed the prosocial learning task^19^ (see later), alongside three other experimental tasks (to be reported elsewhere) and a range of questionnaires (see later). Compensation included a payment of £10 per hour plus expenses, as well as an additional payment of up to £5 depending on the number of points participants earned for themselves during the task. They were also told the number of points that they earned in the prosocial condition would result in a bonus payment of up to £5 for the other participant (see later). No participant reported that they did not believe their choices impacted another person. All participants provided written informed consent, and ethical approval, including for the mild deception about Player 2, was granted by the Oxford University Medical Sciences Inter Divisional Research Ethics Committee and National Health Service Health Research Authority Ethics Ref. 18/LO/2152.

#### Reinforcement learning task

Participants completed a probabilistic reinforcement-learning task in which they learnt to obtain rewards (points) in three recipient conditions: for themselves (‘self’), for another participant (‘other’; prosocial condition) and for neither person (‘no one’; neither participant is rewarded, control condition). Participants learnt about two unique symbols in each block, with new stimuli introduced at the start of each block. Each trial presented one symbol associated with a high (75%) probability of reward, and one symbol associated with a low (25%) probability of reward (Fig. 1C). The two symbols were randomly allocated to either side of the screen and were selected by pressing the corresponding left or right arrow key. The outcome of each trial was either earning 0 or 100 points. After selecting a symbol, participants were shown whether they earned points or not, allowing them to learn over time which symbol maximized rewards (see the Supplementary material for task instructions). There were three blocks of 16 trials for each recipient, resulting in 48 self trials, 48 other trials and 48 no one trials, or 144 trials across six blocks in total. Blocks were pseudo-randomised such that the same recipient condition never appeared consecutively, and participants were randomly assigned to one of six possible order combinations. Points earned were translated into an additional payment for the participant in the self blocks and into a bonus payment for the other participant (who was actually a confederate researcher, see ‘Role assignment’ section) in the other condition. In contrast, in no one blocks, points were displayed but were not converted into a monetary reward for anyone. As such, this condition was used to control for the participants’ general ability to learn probabilistic associations in the absence of monetary reward. Each block started with an instruction screen indicating the recipient condition [‘Play for (you/confederate name/no one)’] for 2000 ms. Subsequently, two abstract symbols (letters from the Agathodaimon font) were presented on either side of a fixation cross. Participants had 3000 ms to select one symbol by pressing the left or the right arrow key. If they failed to respond, the word ‘missed’ appeared in red. Otherwise, the selected option was shown (300 ms), followed by a fixation delay (2500 ms) and the corresponding outcome (either 100 points or 0 points; 800 ms). Another fixation period (1000 ms) preceded the next trial, which showed the same two symbols again. All screens except fixations showed the recipient condition ‘(you/confederate name/no one)’. We used Presentation v17 to present the stimuli (Neurobehavioral Systems, https://www.neurobs.com/).

#### Role assignment

To ensure participants believed that the rewards they gained in the prosocial condition truly benefitted another individual, we performed a commonly used role-assignment procedure^19,35^ (Fig. 1D). This involved anonymously introducing the participant to another participant (who was in fact a confederate of the experimenter) and randomly allocating them the roles of Player 1 or Player 2. Participants were informed that Player 1 would perform tasks with outcomes affecting themselves as well as Player 2, while Player 2’s outcomes would only affect themselves. While our procedure ensured that study participants were in fact always assigned the role of Player 1 (see Supplementary material), this manipulation eliminated the potential influence of reciprocity and social preferences on our participants’ behaviour.^36^ A paid confederate researcher played the role of the other participant to avoid cancelling testing sessions if a dyad member missed their appointment. Study participants were only informed about this once data collection was complete to prevent them from sharing information that might compromise the belief in the reality of Player 2 with other potential participants. Participants completed a standardized debriefing questionnaire after the task, and none reported disbelieving the presence of another real player.

### Questionnaires

Participants completed the Apathy Motivation Index,^33^ Beck Depression Inventory,^34^ Questionnaire of Cognitive and Affective Empathy^31^ and Trail Making Test.^32^ See Supplementary material for further details about each questionnaire.

### Statistical analysis

Data were analysed using R^37^ (version 4.2.2), with R Studio^38^ (version 2021.09.1). Behavioural data and computational parameters (see later) were analysed using (generalized) linear mixed-effects models (LMM; *glmmTMB* function, glmmTMB R package^39^ v1.1.8; see Supplementary material for more detail). *Post hoc* analyses relied on the contrasts of estimated marginal means (*contrast* and *emmeans* functions, emmeans R package^40^ version 1.8.4.1). *Post hoc P*-values were corrected for all presented contrasts using the false discovery rate (FDR) method. Simple contrasts between groups used independent parametric (*t*-test) or non-parametric (Wilcoxon two-sided signed rank test) comparisons. Lastly, we calculated Bayes Factor (BF_01_) for non-significant results using paired and independent Bayesian *t*-tests with default priors (*ttestBF* function, BayesFactor package^41^ v0.9.12.4.6). This computed BF_10_, the Bayes Factor comparing the probability of the data under the alternative hypothesis to that under the null. To facilitate interpretation, we calculated and interpreted BF_01_ (the inverse of BF_10_), representing the probability of the data under the null relative to the alternative hypothesis. Based on the Jeffreys classification scheme, a BF_01_ between 3 and 10 indicated ‘substantial evidence’ in favour of the null hypothesis, whereas a BF_01_ falling between 1 and 3 were labelled ‘anecdotal evidence’, suggesting that that data were unable to distinguish between hypotheses.^42-44^

### Computational modelling

**Figure 2:**
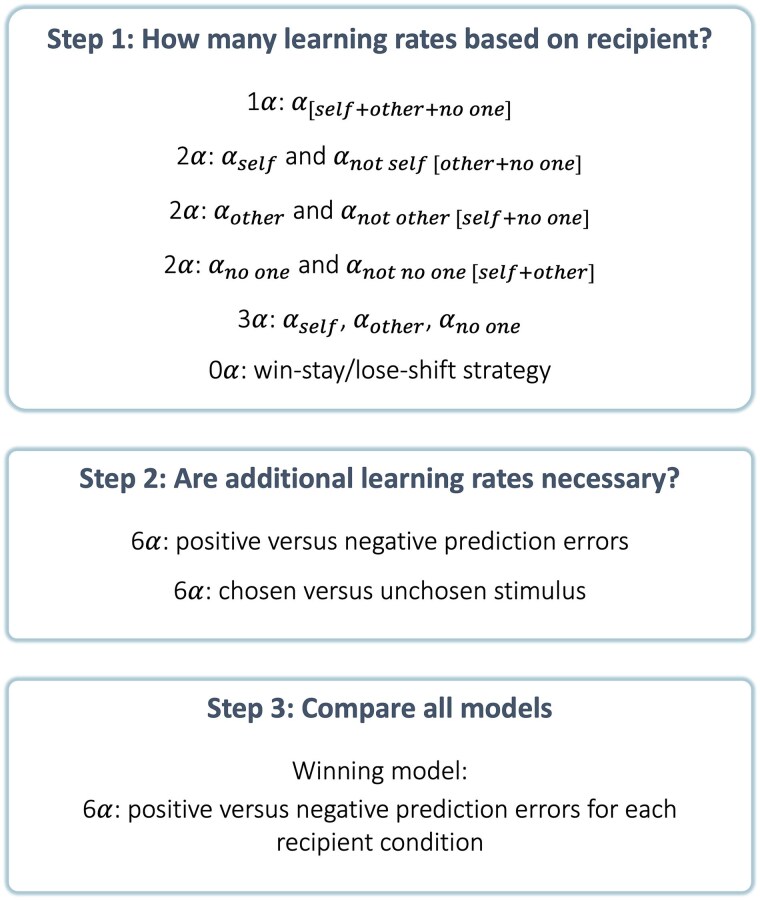
**Model space construction process.** Based on previous work, the first step in building the model space for this study was to determine the number of learning rates necessary based on recipient (self, other, no one). At this stage, we also built a model without any learning rate parameters to test whether participants were using a win-stay/lose-shift strategy. Secondly, we tested whether additional learning rate parameters were necessary to differentiate between positive and negative prediction errors, or between chosen and unchosen stimuli. For all these model variations, we built a version with only one temperature parameter, and a version with three temperature parameters based on recipient condition (self, other, no one). Upon comparing all models, the winning model had six learning rates (self, other, no one for positive prediction errors, and self, other, no one for negative prediction errors) and one temperature parameter.

We modelled trial-by-trial choice behaviour with a range of plausible models representing different explanations of prosocial reinforcement learning. Building on previous work,^28,45,46^ these tested whether the same or different learning rate (α) parameters were needed for the self, other and no one conditions, for positive and negative prediction error trials, and for chosen and unchosen stimuli (Fig. 2). In addition, we tested whether the decision noise (β) parameter varied between recipient conditions, leading to a total of 13 models (see Supplementary material). We fit these models to the choice data in MATLAB^47^ using an iterative maximum a posteriori (MAP) approach, in line with previous studies of reinforcement learning.^28,48,49^ Models were compared based on log model evidence, exceedance probability and integrated Bayesian information criterion, and we used simulated data to establish parameter recovery of the winning model based on our trial schedule^19,28^ (Supplementary Fig. 2). See the Supplementary material for more detail on model fitting, model comparison and parameter recovery.

### Voxel-based lesion-symptom mapping

To examine whether specific vmPFC subregions were causally involved in the behavioural differences in prosocial and self-benefitting reinforcement learning we observed between groups, we conducted VLSM analyses for (i) positive prediction error (PE) learning rates; and (ii) negative PE learning rates; for each, examining (a) other-no one difference; (b) other-self difference; and (c) self-no one difference. Patients’ lesion maps were mirrored and binarized, creating symmetrical masks to enhance statistical power and account for the absence of prior hypotheses regarding laterality.^50,51^ Only voxels with damage in at least five patients were included in the VLSM (Supplementary Fig. 3). *P*-values were generated using permutation-based threshold-free cluster enhancement^52-54^ with 5000 permutations and received Bonferroni correction for multiple comparisons. For visualization, we applied binarized masks of significant areas to *t*-values and plotted regional damage extent against the relevant learning rate differences (see Supplementary material for full details of the VLSM analyses).

Regions of subgenual anterior cingulate cortex were labelled using anatomical masks developed by Palomero-Gallagher and colleagues,^55^ while other parts of vmPFC were labelled using the fourth edition of the *Atlas of the Human Brain*^56^ (https://www.thehumanbrain.info/).

## Results

Twenty-eight patients with focal vmPFC lesions (mean age = 57.94; 15 females) and two control groups took part: 21 lesion control patients with damage to brain areas excluding vmPFC (LC; mean age = 56.33; 13 females) and 124 healthy controls with no brain damage (HC; mean age = 52.94; 71 females). The three groups were carefully matched, with no difference in age, gender or self-reported empathy. In addition, the vmPFC group did not differ from either control group in cognitive ability or levels of apathy. The lesion groups also did not significantly differ from each other in depression (see ‘Materials and methods’ section and Supplementary Table 1).

### vmPFC damage impairs accuracy when learning to obtain rewards for others

**Figure 3:**
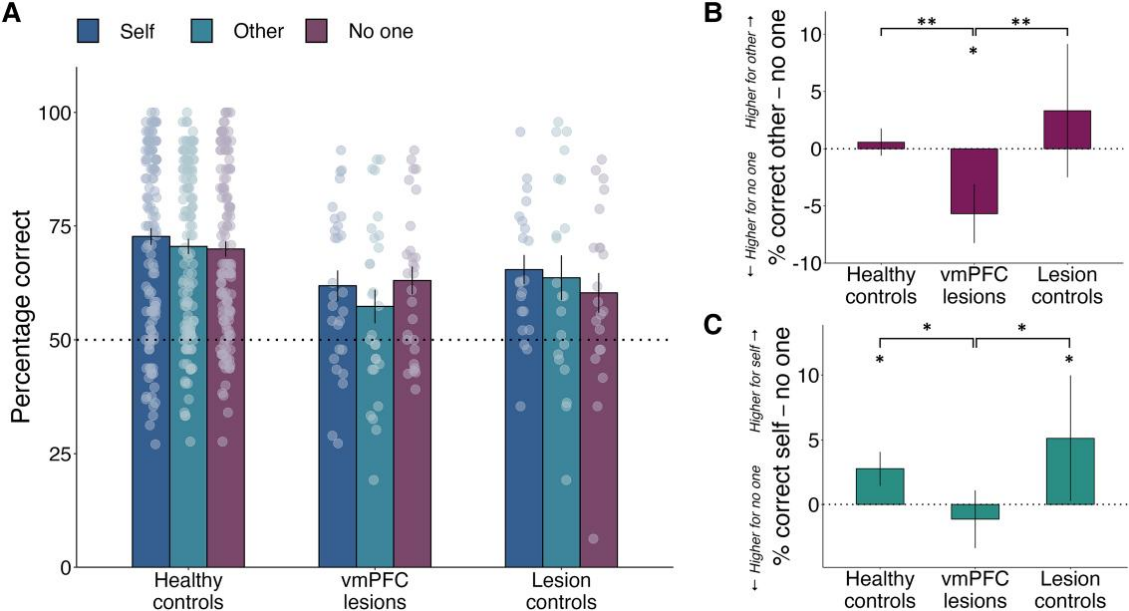
**Damage to vmPFC disrupts the ability to learn to benefit others and self over no one**. (**A**) Unlike both control groups, patients with vmPFC lesions had lower accuracy when learning to benefit another person compared to no one and similar accuracy for self and no one (see Supplementary Tables 2 and 3). Graph displays the percentage correct in each group for each recipient condition (self: dark blue, other: light blue, no one: purple), and dots represent individual data points. (**B**) vmPFC patients are less accurate for other than for no one. (**C**) Both healthy controls and lesion controls show a self-benefitting advantage as they are more accurate for self over no one, a pattern absent after vmPFC damage. Significance reflects the results of a generalised linear mixed effects model analysing the participants’ trial-by-trial binary decisions (i.e. choosing the symbol associated with a low or high probability of reward). Higher-level significance brackets represent significant recipient-by-group interactions, and lower-level asterisks denote within-group differences found in *post hoc* comparisons (FDR-corrected). Error bars represent standard errors of the mean. *n* = 173 (124 healthy controls, 28 patients with ventromedial prefrontal cortex damage, 21 with damage elsewhere). *P < 0.05, **P < 0.01. FDR = false discovery rate; vmPFC = ventromedial prefrontal cortex.

Our first analysis examined how accurate participants were when learning to obtain rewards for themselves, for another anonymous participant, or when no one benefitted (control condition). Accuracy was quantified as the frequency of choosing the high probability symbol, regardless of the outcome subsequently received. We tested whether accuracy differed between the three groups (HC, vmPFC and LC) across trials and conditions using a generalised linear mixed effects model (GLMM; ‘Materials and methods’ section, Fig. 3A, Supplementary Fig. 4 and Supplementary Table 2). We used *post hoc* contrasts between pairs of recipients within each group to interpret the direction of Recipient × Group interactions.

Patients with vmPFC damage performed worse when learning to obtain rewards for another person than for no one [odds ratio (OR) = 0.78, standard error (SE) = 0.07, *Z*-ratio = −2.89, FDR-adjusted *P* = 0.017; Fig. 3B and Supplementary Table 3], whereas HC were equally accurate for other and no one (OR = 1.03, SE = 0.05, *Z*-ratio = 0.74, FDR-adjusted *P* = 0.55, BF_01_ = 7.00, substantial Bayesian evidence in favour of the null), and lesion controls showed no significant difference (OR = 1.17, SE = 0.11, *Z*-ratio = 1.63, FDR-adjusted *P* = 0.15, BF_01_ = 2.98, anecdotal evidence for the null). This was illustrated by significant group-by-recipient interactions between patients with vmPFC lesions and both control groups [vmPFC versus HC: OR (95% confidence interval, CI) = 0.76 (0.63, 0.91), *Z* = −2.91, *P* = 0.004; vmPFC versus LC: OR (95%CI) = 0.67 (0.52, 0.86), *Z* = −3.13, *P* = 0.002]. Interestingly, a follow-up *t*-test additionally revealed that vmPFC patients were unable to learn significantly above chance when rewarding others [mean = 57%, *t*(27) = 2.0, *P* = 0.057, BF_01_ = 0.91, anecdotal evidence against the null]. In contrast, accuracy was significantly higher than 50% in all other conditions and for all other groups [vmPFC: all *t*(27) > 3.5, all *P ≤* 0.001; HC: all *t*(123) > 12.0, all *P* < 0.001; LC: all *t*(20) > 2.4, all *P* < 0.028].

In addition, patients with vmPFC damage were equally accurate when learning to benefit themselves compared to no one (OR = 0.96, SE = 0.08, *Z*-ratio = −0.50, FDR-adjusted *P* = 0.65, BF_01_ = 3.61, substantial Bayesian evidence for the null; Fig. 3C). In contrast, both control groups displayed a self-benefitting advantage as they performed significantly better for self compared to no one (HC: OR = 1.17, SE = 0.05, *Z*-ratio = 3.44, FDR-adjusted *P* = 0.005; LC: OR = 1.27, SE = 0.13, *Z*-ratio = 2.46, FDR-adjusted *P* = 0.035; Group × Recipient interaction, vmPFC versus HC: OR = 0.82, SE = 0.08, *Z*-ratio = −2.02, *P* = 0.043; Group × Recipient interaction, vmPFC versus LC: OR = 0.75, SE = 0.10, *Z*-ratio = −2.18, *P* = 0.029).

Overall, these findings reveal that vmPFC damage led to lower accuracy for prosocial learning and to the absence of the typical preference to benefit self over no one.

### Learning rates depend on valence and reward recipient

Next, we built computational reinforcement learning models to precisely quantify how participants learnt in the task. Our model space included models with different learning rates (α parameters), which indicate the extent to which individuals update their estimate of how rewarding an option is following each outcome (reward or no reward). The models also estimated temperature parameters (β), indexing variability in following the value of each option. We fitted and compared the models using a hierarchical expectation maximization approach^48,49^ and selected the best model based on exceedance probability, representing the chance that the participant data were generated by each model. Our model space allowed variation of learning rates and temperature parameters for each recipient,^19^ for positive and negative prediction errors (PEs) and for chosen and unchosen options. In addition, we examined a control model that evaluated whether participants implemented a simple win-stay lose-shift strategy. This resulted in 13 candidate models constructed iteratively (see the ‘Materials and methods’ section for full details).

In all three groups, the best fitting model had separate learning rates for positive and negative PEs for each recipient, and a single temperature parameter (model ix—6*α*1*β*: *α*_self positive PE_, *α*_self negative PE_, *α*_other positive PE_, *α*_other negative PE_, *α*_no one positive PE_, *α*_no one negative PE_, *β*). Positive PEs occurred when participants experienced the outcome they expected or a better outcome, and negative PEs when they experienced a worse outcome than expected. In addition to having the highest exceedance probability in all three groups (HC: 100%, vmPFC: 96.59%, LC: 98.09%), the winning model also showed the highest log model evidence and lowest integrated Bayesian information criterion (Supplementary Table 4). We additionally showed strong parameter recovery for the winning model (Supplementary Fig. 2), and that simulated data based on this model closely matched the participant choices (Supplementary Fig. 5). The temperature parameter β did not significantly differ across groups [vmPFC versus HC: OR (95%CI) = 0.81 (0.64, 1.00), SE = 0.10, *Z* = −1.80, *P* = 0.07, BF_01_ = 1.14, anecdotal evidence for the null; vmPFC versus LC: OR (95%CI) = 0.84 (0.60, 1.10), SE = 0.14, *Z* = −1.10, *P* = 0.28, BF_01_ = 2.25, anecdotal evidence for the null; HC versus LC: OR (95% CI) = 1.00 (0.80, 1.40), SE = 0.14, *Z* = 0.26, *P* = 0.79, BF_01_ = 3.99, substantial evidence for the null; Supplementary Fig. 6].

### vmPFC damage causes aberrant learning rates after positive PEs for others compared to neither person

**Figure 4:**
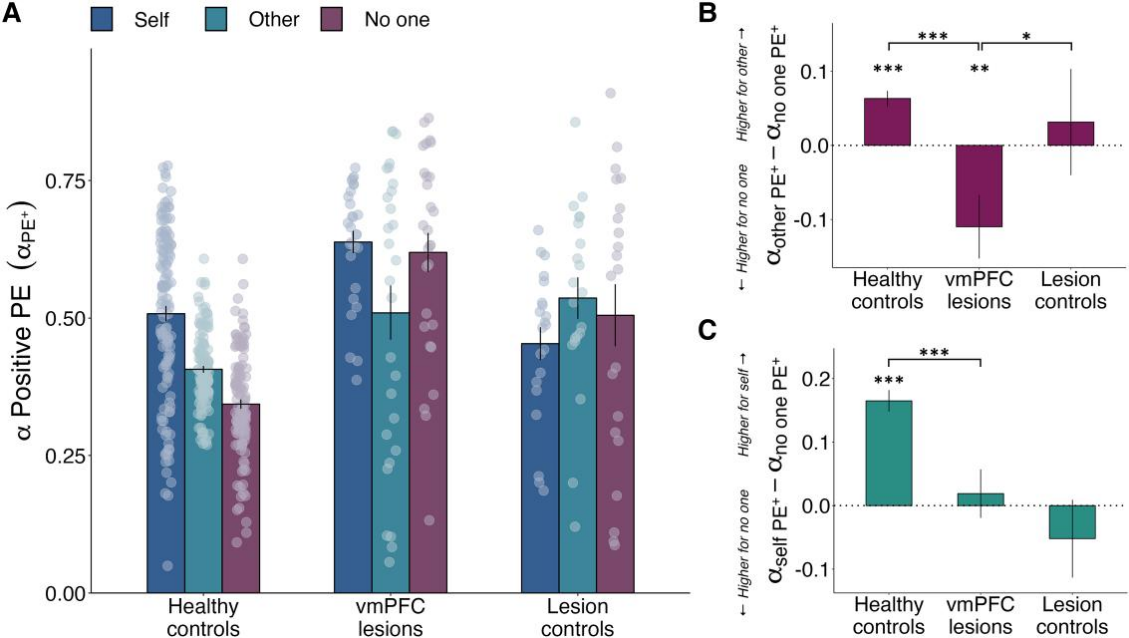
**Damage to vmPFC disrupts learning from positive prediction errors when learning to benefit other and self over no one**. (**A**) Unlike both control groups, vmPFC patients had lower positive PE learning rates when learning to benefit other over no one (see Supplementary Tables 5 and 6). Patients with vmPFC damage also showed similar learning rates for positive PEs for self and no one. Graph displays learning rates for positive PEs in each group for each recipient condition (self: dark blue, other: light blue, no one: purple), and dots represent individual subjects. (**B**) vmPFC patients have higher positive PE learning rates for no one than for other. (**C**) Healthy controls have higher learning rates for positive PEs for self over no one, a pattern absent after vmPFC damage. Significance reflects the results of a generalised linear mixed effects model analysing the participants’ learning rates for positive PE trials (i.e. trials where they obtained an outcome equally as good or better than they expected). Higher-level significance brackets represent significant recipient-by-group interactions, and lower-level asterisks denote within-group differences found in *post hoc* comparisons (FDR-corrected). Error bars represent standard errors of the mean. *n* = 173 (124 healthy controls, 28 patients with ventromedial prefrontal cortex damage, 21 with damage elsewhere). *P < 0.05, ***P < 0.001. FDR = false discovery rate; PE = prediction error; vmPFC = ventromedial prefrontal cortex.

We compared learning rates for positive PEs between groups and conditions with a GLMM (Fig. 4A and Supplementary Table 5). In line with the pattern of results for accuracy, we focused on the differences between the control condition (no one) and the self and other conditions, and how these differences varied across groups. Patients with vmPFC damage displayed atypical learning rates for positive PEs when learning to benefit others relative to no one, compared to both control groups [Group × Recipient interaction, vmPFC versus HC: OR (95%CI) = 0.84 (0.78, 0.91), *Z* = −4.36, *P* < 0.001; Group × Recipient interaction, vmPFC versus LC: OR (95%CI) = 0.87 (0.78, 0.97), *Z* = −2.58, *P* = 0.010; Fig. 4B]. Specifically, patients with vmPFC damage had lower learning rates for positive PEs when another person benefitted compared to when no one benefitted (OR = −0.11, SE = 0.04, *t*-ratio = −3.06, FDR-adjusted *P* = 0.004), whereas healthy controls had higher positive PE learning rates for other than no one (OR = 0.06, SE = 0.02, *t*-ratio = 3.71, FDR-adjusted *P* < 0.001) and lesion controls showed no difference (LC OR = 0.03, SE = 0.04, *t*-ratio = 0.76, FDR-adjusted *P* = 0.50, BF_01_ = 3.03, substantial Bayesian evidence for the null; see Supplementary Table 6).

Next, we evaluated whether vmPFC patients’ lack of self-benefitting advantage extended to learning rates. Indeed, patients with vmPFC damage had similar learning rates for positive PEs when benefitting self and no one (OR = 0.02, SE = 0.04, *t*-ratio = 0.53, FDR-adjusted *P* = 0.60, BF_01_ = 3.39, substantial Bayesian evidence for the null; Fig. 4C). In contrast, healthy controls had significantly higher learning rates for positive PEs for self than for no one (OR = 0.16, SE = 0.02, *t*-ratio = 9.67, FDR-adjusted *P* < 0.001; Group × Recipient interaction, vmPFC versus HC: OR = −0.15, SE = 0.04, *t*-ratio = −3.67, *P* < 0.001). However, vmPFC patients did not significantly differ from lesion controls (Group × Recipient interaction, vmPFC versus LC: OR = 0.07, SE = 0.05, *t*-ratio = 1.29, *P* = 0.20) as the lesion control group did not significantly differ in positive PE learning rates for self and no one (OR = −0.05, SE = 0.04, *t*-ratio = −1.25, FDR-adjusted *P* = 0.25, BF_01_ = 2.53, anecdotal evidence for the null). No other comparisons showed effects on learning rates for positive PEs that were specific to the vmPFC group compared to controls (Supplementary Tables 5 and 6).

These findings suggest vmPFC damage led to aberrant learning rates for positive PEs when learning to obtain rewards for another person and for oneself, relative to when no one benefitted (control condition).

### vmPFC damage causes aberrant learning rates for negative PEs for others compared to self

**Figure 5:**
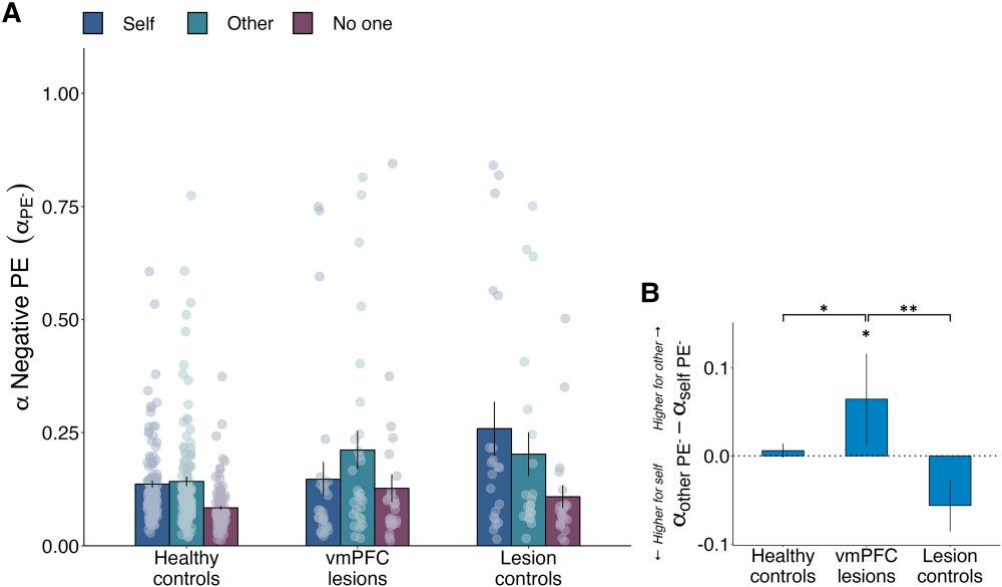
**Damage to vmPFC disrupts learning from negative prediction errors when learning to benefit others**. (**A**) Unlike both control groups, vmPFC patients had higher negative PE learning rates when learning to benefit other over self (see Supplementary Tables 7 and 8). Graph displays learning rates for negative PEs in each group for each recipient condition (self: dark blue, other: light blue, no one: purple), and dots represent individual data points. (**B**) vmPFC patients have higher negative PE learning rates for other than for self. Significance reflects the results of a generalised linear mixed effects model analysing the participants’ learning rates for negative PE trials (i.e. trials where they obtained a worse outcome than they expected). Higher-level significance brackets represent significant recipient-by-group interactions, and lower-level asterisks denote within-group differences found in *post hoc* comparisons (FDR-corrected). Error bars represent standard errors of the mean. *n* = 173 (124 healthy controls, 28 patients with ventromedial prefrontal cortex damage, 21 with damage elsewhere). *P < 0.05, **P < 0.01. FDR = false discovery rate; PE = prediction error; vmPFC = ventromedial prefrontal cortex.

Next, we analysed recipient and group differences in learning rates for negative PEs using a GLMM (Fig. 5A and Supplementary Table 7). We observed differences after vmPFC damage when benefitting other relative to self, compared to both control groups [Group × Recipient interaction, vmPFC versus HC: OR (95%CI) = 1.06 (1.00, 1.12), *Z* = 2.11, *P* = 0.035; Group × Recipient interaction, vmPFC versus LC: OR (95%CI) = 1.13 (1.05, 1.22), *Z* = 3.16, *P* = 0.002; Fig. 5B]. Specifically, patients with vmPFC lesions showed higher learning rates for negative PEs for other than for self (OR = 0.06, SE = 0.03, *t*-ratio = 2.85, FDR-adjusted *P* = 0.023), whereas healthy controls had similar learning rates for both conditions (OR = 0.01, SE = 0.01, *t*-ratio = 0.50, FDR-adjusted *P* = 0.70, BF_01_ = 6.52, substantial Bayesian evidence for the null; Supplementary Table 8). Lesion controls did not show a significant difference between self and other either, although the contrast was not sensitive enough to show evidence of no difference (OR = −0.06, SE = 0.03, *t*-ratio = −1.95, FDR-adjusted *P* = 0.085, BF_01_ = 2.66, anecdotal evidence for the null). No other comparisons revealed effects on learning rates for negative PEs that were specific to vmPFC patients compared to the control groups (Supplementary Tables 7 and 8).

Thus, vmPFC damage led to aberrant learning rates for negative PEs for other compared to self.

### Damage to sgACC is associated with altered prosocial reinforcement learning from positive PEs

So far, we have shown differences in how damage to vmPFC affected learning to benefit others. However, these results were based on group differences comparing damage across a large portion of vmPFC. Our final analyses therefore focused on investigating whether sub-regions of the vmPFC and broader medial prefrontal cortex were causally and specifically involved in differences in learning to obtain rewards for another person and for oneself. We used lesion mapping to examine where damage was associated with the computational differences found between learning to benefit oneself and another person relative to the control condition, and relative to each other. For each valence learning rate (positive PE and negative PE), we ran VLSM analyses correlating the degree of lesion damage at each voxel with the (i) other-no one difference, (ii) other-self difference and (iii) self-no one difference. Analysing the participants’ difference scores allowed us to analyse prosocial and self-benefitting learning separately while controlling for general probabilistic reinforcement learning abilities, as well as comparatively. Crucially, the overall extent of damage (total lesion size) was not significantly correlated with any of these measures across participants (Supplementary Fig. 1).

VLSM examines whether damage at each voxel predicts a person’s behaviour by constructing a map of *t*-statistics.^57^ We only included voxels where at least five patients had damage^58^ across all 49 patients with lesions (28 with vmPFC damage and 21 with damage elsewhere; Supplementary Fig. 3). While this meant that the voxels tested were mostly restricted to the vmPFC, lesion controls were included in the analyses to control for the general effect of having a brain lesion regardless of location. We used the FSL^52^ to run a permutation-based VLSM with threshold-free cluster enhancement^53,54^ and report significance at *P* < 0.0167 (*P* < 0.05 Bonferroni-corrected across three behavioural regressors; see the ‘Materials and methods’ section).

**Figure 6:**
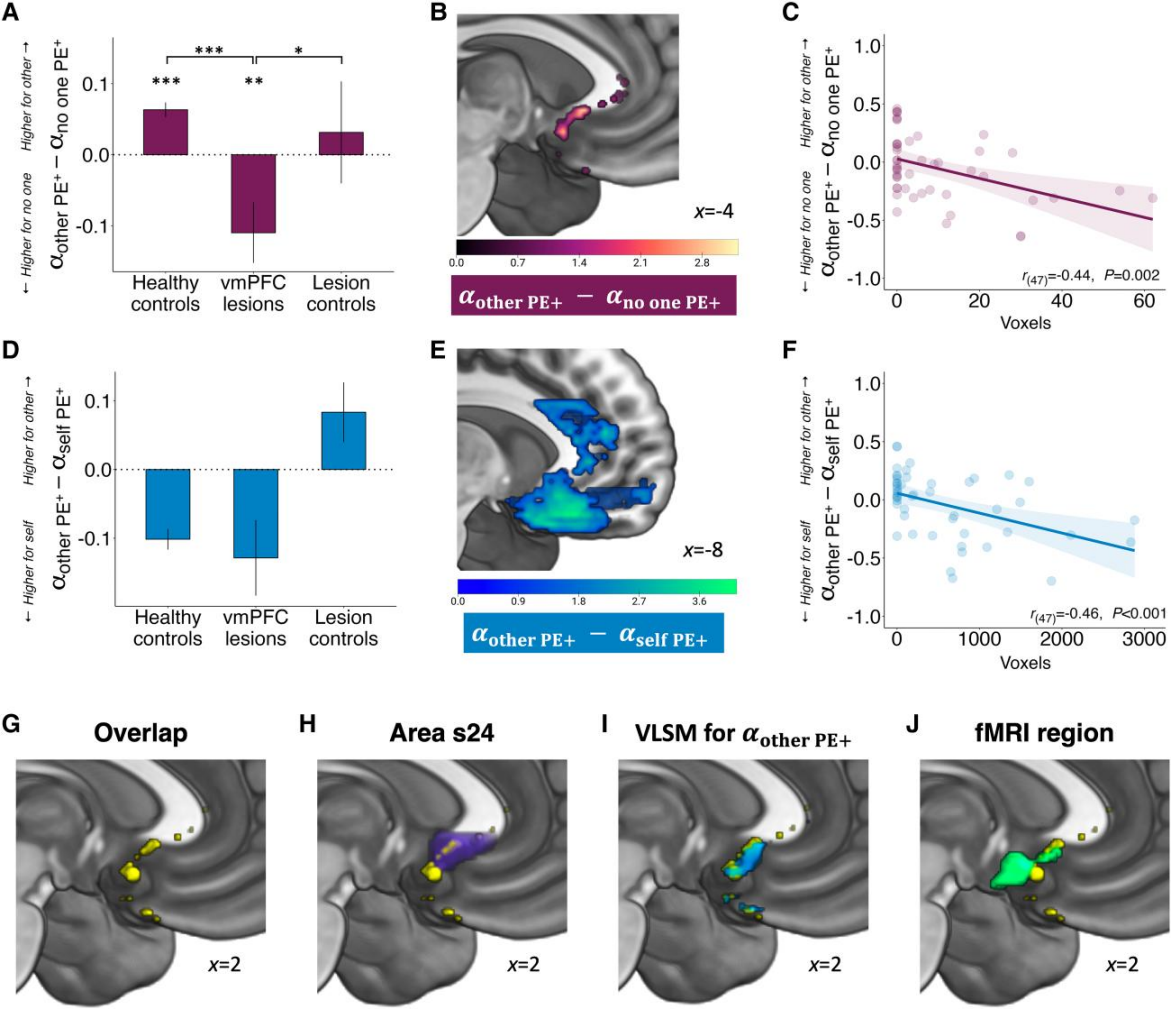
**Damage to sgACC is specifically associated with aberrant prosocial learning rates from positive prediction errors.** (**A**) Unlike both control groups, vmPFC patients had higher positive PE learning rates for no one than for other. (**B**) Permutation-based, whole-brain, non-parametric VLSM reveals that damage to sgACC is associated with the difference between other and no one learning rates for positive PEs. Specifically, this result was located within area s24 (peak ±4, 20, −4). (**C**) A negative association between the difference in positive alphas for other-no one against the extent of damage in the area identified in the relevant VLSM analysis. Note that this graph is provided for visualization and interpretation of the VLSM result and is not intended for quantitative analysis. (**D**) vmPFC patients and healthy controls had higher positive PE learning rates for self than for other. (**E**) Permutation-based, whole-brain, non-parametric VLSM reveals that damage to a large part of the vmPFC is associated with the other-self difference in learning rates for positive PE (peak 8, 32, −20). (**F**) The difference in positive alphas for other-self was negatively associated with the extent of damage in the area identified in the relevant VLSM analysis. Note that this graph is provided for visualization and interpretation of the VLSM result and is not intended for quantitative analysis. (**G**) The conjunction area between the other-no one and the other-self VLSM results (yellow) is (**H**) partly located within area s24 in sgACC (purple). (**I**) A follow-up analysis revealed that damage in a closely matching region disrupted prosocial learning rates for positive PEs (blue). (**J**) The conjunction area also overlapped with a region found to encode prosocial PEs in a functional MRI study^19^ (green). Error bars in **A** and **D** represent standard errors of the mean. *n* = 49 (28 patients with ventromedial prefrontal cortex damage, 21 with damage elsewhere). *P < 0.05, **P < 0.01, ***P < 0.001. PE = prediction error; sgACC = subgenual anterior cingulate cortex; VLSM = voxel-based lesion mapping; vmPFC = ventromedial prefrontal cortex.

For the analysis of learning rates for positive PEs, we first investigated which subregions were associated with the difference between other and no one (Fig. 6A). Damage to the sgACC (Fig. 6B), putatively in areas s24^55,59^ and 32,^59,60^ was associated with a decrease in the other-no one difference (Fig. 6C and Supplementary Fig. 7A and B). In other words, lesions to this area reduced the integration of rewarding feedback during learning when another person benefitted, relative to when no one benefitted. Next, we evaluated which parts of vmPFC encoded learning rates for positive PEs for other-self (Fig. 6D) and found that an overlapping region in sgACC was associated with a decrease in the other-self difference (Fig. 6E and F and Supplementary Fig. 7C and D). For visualization, we combined these two results and constructed a map of their overlap (Fig. 6G), which showed commonality in area s24 of the sgACC (Fig. 6H). As an exploratory follow-up analysis, we checked whether lesions within the same area disrupted prosocial learning rates for positive PEs only (*P* < 0.05, uncorrected). Indeed, damage to the same portion of s24 in the sgACC was associated with lower learning rates for others specifically (Fig. 6I). Finally, we evaluated how this conjunction area corresponded to the area previously found to code prosocial PEs in human fMRI studies of reinforcement learning.^19^ Strikingly, there was again close correspondence between these regions (Fig. 6J).

Whereas damage to the sgACC was specific to the other-no one difference and overlapped with a region for the other-self difference, we also identified additional areas that encoded the other-self difference. Indeed, a more lateral portion of vmPFC putatively in area 13 was negatively associated with the other-self learning rate difference for positive PEs (Fig. 6E). In other words, patients with damage here had lower learning rates for other relative to self. Finally, we examined whether some parts of vmPFC were associated with the self-no one difference in learning rates for positive PEs. Here, damage to more lateral parts of vmPFC, including area 13, was associated with higher learning rates for self relative to no one (Supplementary Fig. 8).

### ACCg and sgACC damage is associated with altered prosocial reinforcement learning from negative PEs

**Figure 7:**
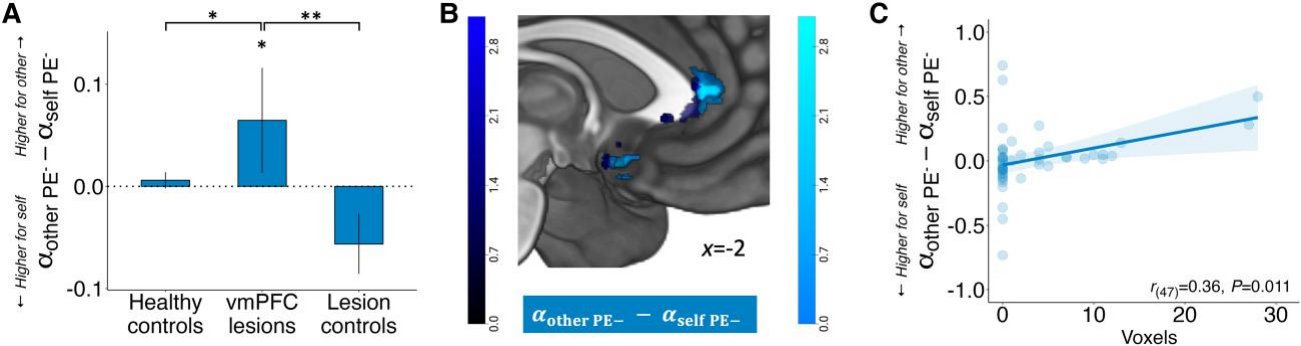
**Damage to ACCg and sgACC is associated with aberrant prosocial learning rates for negative prediction errors**. (**A**) Unlike both control groups, vmPFC patients had higher negative PE learning rates for other than for self. Error bars represent standard errors of the mean. (**B**) Permutation-based, whole-brain, non-parametric VLSM reveals that damage to ACCg and sgACC is associated with the difference between other and self learning rates for negative PEs. Dark blue regions survived correction at *P* < 0.0167 both in ACCg (±2, 34, 8) and sgACC (8, 6, −16; ± 4, 10, −10). (**C**) A positive association between the difference in negative alphas for other-self and the extent of damage in the area identified in the relevant VLSM analysis. Note that this graph is provided for visualization and interpretation of the VLSM result and is not intended for quantitative analysis. *n* = 49 (28 patients with ventromedial prefrontal cortex damage, 21 with damage elsewhere). *P < 0.05, **P < 0.01. ACCg = anterior cingulate cortex gyrus; PE = prediction error; sgACC = subgenual anterior cingulate cortex; VLSM = voxel-based lesion mapping; vmPFC = ventromedial prefrontal cortex.

In a second VLSM analysis, we assessed the correlation between damage at each voxel with the differences in learning rates for negative PEs between self, other and no one. Here, parts of the ACCg (putatively in areas 24a′/b′) as well as the sgACC (putatively areas s24 and 25) encoded the other-self difference (Fig. 7 and Supplementary Fig. 9). In other words, damage to ACCg and sgACC led to an increased integration of negative PEs (here, information about neutral outcomes) when learning to obtain prosocial rewards relative to self-benefitting rewards. Damage to area 14c^59^ was associated with a decrease in the self-no one learning rate difference for negative PEs, and there were no voxels where damage was significantly associated with the other-no one difference (see Supplementary Table 9 for full results).

Overall, the VLSM analyses provided converging evidence that damage to a portion of the sgACC previously identified in human fMRI studies specifically disrupts prosocial reinforcement learning from positive and negative PEs. In addition, a part of ACCg, putatively in areas 24a′/b′, encodes the behavioural difference for negative PE learning rates when benefitting others compared to oneself.

## Discussion

Reinforcement learning is a fundamental process whereby humans and other animals attain rewards for themselves and their conspecifics.^61^ Whilst previous neuroimaging in humans has suggested that both types of learning may depend on vmPFC,^19,20,62^ the causal impact is unknown. Using an incentivized reinforcement learning task in a large sample of patients with rare focal vmPFC lesions and two carefully matched control groups, we reveal converging evidence that vmPFC damage disrupts prosocial reinforcement learning. Compared to controls, patients with vmPFC lesions: (i) were less accurate in gaining rewards for another person relative to when no one benefitted; (ii) had lower learning rates from positive PEs for others relative to no one; and (iii) had higher learning rates from negative PEs for others relative to self. Voxel-based lesion mapping revealed that damage to a region of sgACC previously identified in human fMRI^19^ underpins these behavioural effects. In contrast, damage to ACCg was associated with aberrant negative PE learning rates when learning to benefit others relative to oneself. Finally, vmPFC damage was associated with a reduced self-benefitting advantage, with patients displaying equal accuracy and similar learning rates from positive PEs for self and neither. While previous evidence has shown that patients with vmPFC lesions have increased choice variability,^63^ here, the temperature parameter did not significantly differ between groups. Together, our results suggest a causal influence of vmPFC in learning to benefit other people and highlight that distinct subregions, sgACC and ACCg, play critical roles in prosocial learning.

Our findings go beyond previous work in non-human animals and human fMRI^18-20,64-66^ by showing a causal role of sgACC in learning to help others. vmPFC damage led to reduced accuracy as well as atypical learning from PEs when outcomes benefitted another person over oneself or neither individual. Importantly, lesion-symptom mapping showed that damage to sgACC caused these prosocial behaviour differences in a region where activity had previously been found to correlate exclusively with prosocial prediction errors but not self-relevant ones.^19^ Using a similar paradigm, recent research showed that oxytocin selectively modulates the encoding of prosocial PEs in the sgACC.^20^ Since sgACC activity has been associated with other types of social learning, including learning by observing another individual,^67,68^ learning from social feedback^69^ and integrating social advice,^70^ future studies could examine the causality of sgACC and the role of oxytocin in these processes. Interestingly, changes in sgACC structure and function are also involved in the pathophysiology of depression.^71^ In cases where usual lines of treatment have failed, targeting this region with deep brain stimulation has been found to alleviate depression symptoms successfully.^72^ In light of the current findings, it would be interesting to see how such stimulation of the sgACC influences prosocial behaviour and learning in patients with depression.

Damage to ACCg led to aberrant learning rates for negative PEs when benefitting others relative to self. This result supports evidence from human neuroimaging studies that the ACCg signals social information, particularly during reinforcement learning.^19,24,73-75^ In monkeys, early evidence showed that animals with ACCg lesions displayed poorer valuation of social cues,^76^ suggesting an important role for this region in social behaviour. Employing prosocial reinforcement learning paradigms similar to the one used here, further studies have shown that monkey ACCg neurons selectively activate for prosocial rewards^77,78^ and that ACC lesions cause animals to become unable to learn to benefit another monkey over no one.^79^ In conjunction with these findings, our results point to ACCg as a crucial region for prosocial reinforcement learning across species. Novel techniques such as focused ultrasound stimulation^80^ could be used to target the sgACC and ACCg and further reveal their respective roles in prosocial learning. In animals, focused ultrasound stimulation of the medial prefrontal cortex has been shown to alter social behaviour^81^ and reward processing.^82^

The loss of the self-benefitting advantage in patients with vmPFC lesions is consistent with previous studies emphasizing this region’s role in self-benefitting reinforcement learning.^1-3,62^ Nevertheless, another interpretation of this finding could be that it is caused by an impairment in learning from real, as opposed to hypothetical rewards. In healthy adults, Scholl and colleagues have shown that vmPFC activity is associated with an irrational bias towards selecting choice options associated with real self-benefitting monetary rewards rather than hypothetical (foregone) rewards, even when it is disadvantageous.^45^ In the current study, the self condition is associated with a real monetary reward, while the no one condition may be considered hypothetical, as the points obtained do not result in a financial reward for anyone. Accuracy and positive PE learning rates becoming equal for self and no one in patients with focal vmPFC damage is consistent with the bias towards real rewards disappearing.

While studies examining prosocial reinforcement learning have often found that the participants’ choices were best described by a model with separate learning rates for each recipient,^19,28^ our winning model further distinguished between learning rates for positive and negative PEs. Importantly, this model showed the best fit in all the patient groups based on numerous model comparison metrics. Prior research showed that vmPFC may have a critical role in processing valence and suggested that subregions of vmPFC may preferentially encode either positive^7-9^ or negative^10^ outcomes. Here, vmPFC lesions affected learning rates for both positive and negative PEs, and our results suggested that the involvement of some vmPFC subregions may depend on whether learning occurs in a social context rather than on valence. Indeed, sgACC damage led to abnormalities in prosocial learning rates regardless of whether outcomes were more or less rewarding than expected. This supported a recent neuroimaging study finding that sgACC activity is linked to both positive and negative prosocial PEs during reinforcement learning.^64^ Nevertheless, damage to ACCg seemingly only affected prosocial learning rates from negative PEs, suggesting that other regions of vmPFC may have valence-specific functions. These results emphasize the importance of integrating social specificity and valence-based distinctions in computational models of reinforcement learning. This knowledge will ultimately be critical to predicting specific changes in social behaviour after brain damage.^83^

Future studies should assess whether impairments in prosocial learning differ in more complex learning tasks, such as learning about more than two symbols at a time. Using computational models including forgetting rates, this work could assess whether prosocial learning impairments become more pronounced when having to hold items in working memory.

Although the current study offers novel insights, it also has limitations. Both social behaviours and reinforcement learning rely on large networks of interconnected cortical and subcortical regions such as ventral striatum and amygdala.^84^ The current study investigated the location of function within vmPFC, but it would be important for future research to examine how these regions interact during prosocial learning. Secondly, whilst our study included a relatively large group of patients with lesions compared to previous work, lesion mapping analyses could reveal additional areas of specialization using a larger sample. For instance, ventral striatum is thought to process PEs during learning,^22^ and while some of our patients had lesions extending into ventral striatum, we may have needed more to reliably detect the impact of striatum lesions on prosocial reinforcement learning. Relatedly, it is well known that there is considerable variability in the structure of vmPFC between individuals,^85^ so although we identified subregions, we could not confirm the existence of sharp anatomical boundaries. Another limitation of the current study was that it relied on natural lesions caused by vascular or haemorrhagic pathology; therefore, the lesions tended to occur in specific patterns. This leads to correlations among voxels and can bias the estimated locations of particular cognitive functions.^86^ With larger sample sizes, it may be possible to estimate and compensate for this covariance using multivariate analysis.^87^ Additionally, we had no predefined hypothesis about lateralization and thus mirrored patients’ lesions to increase power. However, a recent study found evidence of differential function of more dorsal left and right prefrontal regions in decision-making.^88^ Future studies with larger samples of patients with focal lesions could investigate functional lateralization of vmPFC, particularly sgACC and ACCg, in prosocial learning and decision-making. Lastly, other people’s advice, opinions and preferences greatly influence many of our social behaviours and decisions. Studies have suggested that such social influences on behaviour may also depend on medial prefrontal cortex,^73,89,90^ and it would be important to examine how focal lesions affect the influence of social information on choices.

In conclusion, we found that vmPFC is necessary for learning to benefit others and that distinct subregions make different contributions. Damage to vmPFC led to multiple impairments in learning to benefit other people, with sgACC and ACCg affecting learning from positive and negative prediction errors. In addition, damage to vmPFC more broadly led to a reduced preference to benefit oneself. Our results emphasize the multifaceted functions of vmPFC in reinforcement learning and social cognition.

## Supplementary Material

awaf056_Supplementary_Data

## Data Availability

The code and anonymized data used in this study are available at: https://osf.io/fcq32/.
