# Supplementary material for: Ventromedial prefrontal cortex lesions disrupt learning to reward others
Source: Brain. 2025 Feb 11;148(10):3537–50. doi: 10.1093/brain/awaf056 (PMC12493044; doi:10.1093/brain/awaf056)
Supplement: awaf056_Supplementary_Data [file awaf056_supplementary_data.pdf]

Supplementary Information

Task instructions

“In this experiment you will see a pair of symbols on each trial, and you need to select one of them. You will receive points for some of your choices, which will be converted into money at the end of the experiment, so the more points you get the more extra money you will earn. The two symbols are not the same in terms of how often they give you points: some of the symbols will give you points more often than others. Each symbol has its own meaning, regardless of where it appears on the screen (so left and right are not important) or when it occurs in the task. You will play the task in three conditions, for yourself, for the other participant, and for no one. When you are playing for yourself, you will receive any money you win. When you are playing for the other participant, they will receive any money you win for them. However, the other participant won’t know how much money you earn from them until they leave the experiment (you will leave the scanning centre at different times, and the money you earn for the other participants will be given to them in a sealed envelope), and they do not know that you are performing a task where you could win extra money for them. When you play for no one, neither of you will receive any of the money. Respond using the left and right arrow keys to make your selection. Try to respond as quickly as possible, you have about 2 seconds to make your choice.”

Role assignment

We anonymously introduced each participant to another participant (who was in fact a confederate of the experimenter) and informed them that they would both pick a ball out of a box to randomly allocate them to the role of Player 1 or Player 2. Participants were told that the tasks performed by Player 1 would affect themselves as well as Player 2, while Player 2’s outcomes would only affect themselves. Study participants were in fact always assigned the role of Player 1, but this procedure eliminated any potential influence of reciprocity and social preferences. In addition, we emphasised that Player 2 would be unaware of the tasks performed by Player 1 and that any reward won for them would be given anonymously.

To make it clear that another individual was involved, the participant and confederate were brought to each side of a door by separate experimenters and were instructed to wave to each other while wearing a glove (**Fig. 1D**). To maintain complete anonymity, both individuals were asked to remain silent and only ever saw the other person’s gloved hand. The experimenter then tossed a coin to determine who would pick a ball first and subsequently informed the participants of their assigned roles based on the ball they picked. While most identifying characteristics of Player 2 remained undisclosed to the participant, Player 2 was referred to by a name gender-matched to the participant. This strengthened the realism of prosocial decisions while minimising confounds. No participant reported disbelieving the presence of another real player in the standardised debrief questionnaire completed at the end of the session.

Questionnaires

Apathy Motivation Index
The Apathy-Motivation Index[1] (AMI) was used to assess apathetic traits. This 18-item questionnaire measures individual differences in apathy and motivation and requires participants to rate their agreement with each item on a 5-point Likert scale (0-4). Higher scores indicate higher apathy.

Beck Depression Inventory
We used the 21-item Beck Depression Inventory[2] (BDI) to measure depressive symptoms. Participants indicated their agreement with each item on a 4-point Likert scale (0-3) for each item, with higher scores reflecting higher depression.

Questionnaire of Cognitive and Affective Empathy
The Questionnaire of Cognitive and Affective Empathy[3] (QCAE) is composed of 31 items split into two subscales respectively measuring cognitive and affective empathy. Subjects evaluate item on a four-point scale ranging from "strongly disagree" to "strongly agree."

Trail Making Test
The Trail Making Test[4] (TMT) comprises two sections (A and B), and participants are instructed to complete these as quickly and accurately as possible. TMT-A involves sequentially connecting 25 numbers in ascending order (i.e., 1–2–3–4, etc.). The numbers are randomly positioned on a sheet of paper, serving as a measure of visual attention. In TMT-B, the participants must alternate between numbers (1–13) and letters (A–L) while connecting them (i.e., –A–2–B–3–C, etc.), in order to assess executive function. The score on each part represents the amount of time needed to finish the respective task.

Statistical analysis

Trial-by-trial binary choices were modelled with a binomial generalised LMM (GLMM) containing fixed effects of recipient (self or other), group (vmPFC, HC or LC), trial number, as well as all possible interactions, and a subject-level random intercept. In all models, group and agent were coded using treatment contrasts, with the vmPFC lesion group and the prosocial recipient as reference levels. Trial number was z-scored before being entered into the model. Models of computational parameters ($\alpha_{\text{positive PE}}$ and $\alpha_{\text{negative PE}}$) had fixed effects of recipient, and a subject-level random intercept, as there is only one datapoint per participant per recipient.

The final models for each variable were:

Choice ~ Recipient * Group * Trial Number + (1|ID) with binomial link function

$\alpha_{\text{positive PE}}$ ~ Recipient * Group + (1|ID)

$\alpha_{\text{negative PE}}$ ~ Recipient * Group + (1|ID)

We used the parameters package[5] (model_parameters function) to extract standardised model coefficients (exponentiated to generate odds ratios), their standard errors, and 95% confidence

intervals. P-values were computed based on the Wald z-test, the default in glmmTMB. All other parameters were glmmTMB default.

Computational modelling

We assessed learning rates (α) and decision noise (temperature, β) parameters by comparing a range of models that represented different plausible explanations of prosocial reinforcement learning. First, we used a basic model of reinforcement learning, which includes a single learning rate α and temperature parameter β. In this model, the expected value Q of a stimulus s on the next trial $t + 1$ is computed as the value of this stimulus on the current trial, updated with a prediction error PE scaled by the learning rate α:

$$\mathrm{Q}_{t+1}(s) = \mathrm{Q}_t(s) + \alpha \times PE \qquad (1)$$

Where PE is the discrepancy between the actual outcome received on the current trial r_t and the expected value of the stimulus chosen on this trial:

$$PE = r_t - \mathrm{Q}_t(s) \qquad (2)$$

As such, the learning rate dictates the influence of the prediction error on decision-making.

Expected values are then passed through the Softmax function to obtain the probability of choosing a stimulus on each trial depending on the temperature β:

$$p_t[(s|\mathrm{Q}_t(s))] = \frac{e^{(Q_t(s)/\beta)}}{\sum_{s'} e^{(Q_t(s')/\beta)}} \qquad (3)$$

The temperature parameter β serves as a measure of decision noise, representing whether a participant tends to explore different options or consistently opts for the choice with the highest expected value. A high β implies that decisions appear more random, as all options are equally likely to be selected regardless of their expected value. In contrast, a low β value signifies choosing the option with the highest expected value on all trials.

As outlined in **Fig. 2**, we first sought to determine how many agent-based learning rates were needed to best describe choices in patients with vmPFC damage and healthy controls, with versions including between zero and three learning rates. Previous research using the same task has shown that a model with three learning rates (one α for each recipient condition) best characterised healthy participants decisions compared to models with either one or two learning rates combining recipient conditions.[6] In the current study, we compared these models to a win-stay/lose-shift model, which does not include a learning rate because it does not involve learning contingencies, but rather choosing based only on the most recent outcome. Indeed, using this decision-making strategy implies that choices are completely dependent on the most immediate previous outcome. One would 'stay' with the same stimulus if it just yielded a reward, and 'shift' to another if it had not. We found that models with three learning rates, one for each recipient, best described choices in both groups.

Next, we investigated whether additional learning rates may be necessary in either group. Based on research showing that vmPFC may be involved in valence processing, we constructed candidate models testing whether learning rates were different for positive versus negative

prediction errors. As such, for each agent, these models included two learning rates depending on prediction errors:

$$Q_{t+1}(s) = Q_t(s) + \alpha_{[valence]\ for\ [agent]} \times PE \qquad (4)$$

Additionally, based on research highlighting the importance of hypotheticals and counterfactual learning in reinforcement learning,[7,8] we built models testing whether learning rates might differ for chosen versus unchosen stimuli. For each agent, these models included two learning rates updating the value of each stimulus on each trial:

$$Q_{t+1}(s) = Q_t(s) + \alpha_{[chosen/unchosen]\ for\ [agent]} \times PE_a \qquad (5)$$

For each model family, we also built a version with a single noise parameter, and a version with three noise parameters (one per recipient condition). The final model space comprised the following:

(i) $1\alpha1\beta$: one α for all three recipients and one β for all three recipients
(ii) $3\alpha1\beta$: α_{self}, α_{other}, $\alpha_{no\ one}$, β
(iii) $2\alpha1\beta$: α_{self}, $\alpha_{not\ self[other\ +\ no\ one]}$, β
(iv) $2\alpha1\beta$: α_{other}, $\alpha_{not\ other[self\ +\ no\ one]}$, β
(v) $2\alpha1\beta$: $\alpha_{no\ one}$, $\alpha_{not\ no\ one[self\ +\ other]}$, β
(vi) $3\alpha3\beta$: α_{self}, α_{other}, $\alpha_{no\ one}$, β_{self}, β_{other}, $\beta_{no\ one}$
(vii) $0\alpha1\beta$: no α based on win-stay/lose-shift, β
(viii) $0\alpha3\beta$: β_{self}, β_{other}, $\beta_{no\ one}$
(ix) $6\alpha1\beta$: $\alpha_{self\ positive\ PE}$, $\alpha_{self\ negative\ PE}$, $\alpha_{other\ positive\ PE}$, $\alpha_{other\ negative\ PE}$, $\alpha_{no\ one\ positive\ PE}$, $\alpha_{no\ one\ negative\ PE}$, β
(x) $6\alpha3\beta$: $\alpha_{self\ positive\ PE}$, $\alpha_{self\ negative\ PE}$, $\alpha_{other\ positive\ PE}$, $\alpha_{other\ negative\ PE}$, $\alpha_{no\ one\ positive\ PE}$, $\alpha_{no\ one\ negative\ PE}$, β_{self}, β_{other}, $\beta_{no\ one}$
(xi) $4\alpha1\beta$: $\alpha_{self\ chosen}$, $\alpha_{other\ chosen}$, $\alpha_{no\ one\ chosen}$, $\alpha_{unchosen}$, β
(xii) $5\alpha1\beta$: $\alpha_{self\ chosen}$, $\alpha_{other\ chosen}$, $\alpha_{no\ one\ chosen}$, $\alpha_{other\ unchosen}$, $\alpha_{not\ other\ [self\ +\ no\ one]\ unchosen}$, β
(xiii) $6\alpha3\beta$: $\alpha_{self\ chosen}$, $\alpha_{other\ chosen}$, $\alpha_{no\ one\ chosen}$, $\alpha_{self\ unchosen}$, $\alpha_{other\ unchosen}$, $\alpha_{no\ one\ unchosen}$, β_{self}, β_{other}, $\beta_{no\ one}$

Model fitting

We fit these models to the choice data in MATLAB[9] using an iterative maximum a posteriori (MAP) approach, in line with previous studies of reinforcement learning.[6,10,11] For this, we initialized Gaussian distributions as non-informative priors with an average of 0.1 (plus some noise) and a variance of 100. In the expectation step, we estimated model parameters for each participant through maximum likelihood estimation (MLE), calculating the log-likelihood of their response data given the model under consideration. We subsequently computed the maximum posterior probability estimate based on the participants' actual choices and on the prior probabilities from the Gaussian distribution. During the maximization step, we updated the Gaussian distribution describing the model parameters. These alternating expectation and maximization steps were iteratively performed until either the posterior likelihood converged (defined as a change in posterior likelihood <0.001 between consecutive iterations), or a

maximum of 800 iterations were reached. When 800 iterations were reached, the script looped back and attempted fit the model again using a new starting value. This ensured that all models eventually converged within 800 iterations. Additionally, we transformed bounded free parameters from the Gaussian space into the native model space using link functions, such as a sigmoid function for learning rates. Due to the large difference in group sizes, modelling was run separately for each group.

Model comparison

Based on established procedure,[12] we compared models by computing the Laplace approximation of the log model evidence[13] (LME), where higher values indicated a better fit for the model. These results were then subjected to a random-effects analysis[14] using spm_BMS from Statistical Parametric Mapping (SPM12[15]; update revision number 7771; https://www.fil.ion.ucl.ac.uk/spm/software/spm12/). This analysis produced the exceedance probability, which represents the posterior probability that a given model is the most likely among the set of models considered for the given population. A higher exceedance probability suggests stronger evidence in favour of a particular model, with values over 0.95 strongly supporting the best-fitting model. Lastly, we calculated the integrated BIC (BIC_{int}), which penalises models with numerous parameters, as an additional metric for model fit. Lower BIC_{int} values indicate better performance.

Parameter recovery

We used simulated data to establish that the parameters could be accurately estimated based on our trial schedule.[6,16] Data was simulated using a grid of values to cover the full possible ranges of the seven parameters ($\alpha_{positive\ PE\ self}$, $\alpha_{negative\ PE\ self}$, $\alpha_{positive\ PE\ other}$, $\alpha_{negative\ PE\ other}$, $\alpha_{positive\ PE\ no\ one}$, $\alpha_{negative\ PE\ no\ one}$, β) in the winning model across 4374 simulated agents (α values: 0.2, 0.5, 0.8; β values: 0.01, 0.11, 0.21, 0.31, 0.41, 0.51, all with added noise drawn from a normal distribution * 0.5). We fit the simulated data using the MAP approach applied to data from the participants and created a confusion matrix of the correlations between simulated and fitted parameter values (**Supplementary Fig. 2**).

Voxel-based lesion-symptom mapping (VLSM)

To examine whether specific vmPFC subregions were causally involved in prosocial and self-benefitting reinforcement learning, we conducted VLSM analyses for 1) positive PE learning rates and 2) negative PE learning rates, for each examining i) other-no one difference, ii) other-self difference, and iii) self-no one difference. These analyses therefore examined the specific subregions of vmPFC where damage was causing the behavioural differences we observed between groups. We used the learning rate parameters specifically, in line with our computational neurology approach, to separately test hypotheses about positive and negative learning rates.

We quantified these variables for each participant by calculating individual learning rates (see Computational modelling), then subtracting i) the learning rate for no one from the learning rate for other, ii) the learning rate for self from the learning rate for other, and iii) the learning rate for no one from the learning rate for self. Because the no one condition measured general

probabilistic associative learning, subtracting learning rates for this condition from our conditions of interest (other; self) allowed us to obtain more precise assessments of the effect of reward recipient for each individual, while controlling for their general associative learning abilities. Altogether, this led to three difference scores for positive learning rates and three difference scores for negative learning rates for each participant.

We used the *randomise* function within the FMRIB software library (FSL)[17] to conduct permutation-based VLSM analyses.[18,19] We mirrored and binarized patients' lesion maps, creating symmetrical masks to enhance statistical power and account for the absence of prior hypothesis regarding laterality. To mirror lesion maps, we collapsed data across the two hemispheres by summing the voxel values in SPM12,[15] producing a symmetrical representation. Mirrored lesion maps were then binarized by assigning a value of 1 to voxels with summed values ≥1, while voxels with values of 0 remained unchanged. Consistent with prior work,[20,21] this approach ensured a consistent binary mask across hemispheres. The inclusion of voxels in the VLSM required that at least five patients had damage in each voxel to some degree (**Supplementary Fig. 3**). While this meant that the voxels tested were mostly restricted to the vmPFC, lesion controls were included in the analyses to control for the general effect of having a brain lesion regardless of location. Following FSL specifications for the nature of our design, each regressor was ranked and then demeaned. *P*-values were generated using permutation-based threshold-free cluster enhancement (TFCE) in randomise with 5000 permutations and FSL's default TFCE settings, optimised for this type of data.[17,19] Permutation testing involved multiple repetitions of the same analysis with the data randomly shuffled, calculating voxel-wise *p*-values to capture the likelihood that the real, observed effect could be attributed to random noise. This approach aligns more closely with the data's inherent characteristics, relies on fewer assumptions than alternative methods, and can be combined with the benefits of TFCE.[19]

For positive PE and negative PE learning rates, we applied a Bonferroni correction for multiple comparisons across the three difference scores (other-no one, other-self, and self-no one; $p<0.0167$) to the uncorrected maps from the permutation-based TFCE results. For visualisation, we applied binarized masks of the significant areas from each analysis to the *t*-values. We also extracted the extent of damage in these regions for each participant to plot against the relevant learning rate difference scores.

Regions of subgenual anterior cingulate cortex were labelled using anatomical masks developed by Palomero-Gallagher and colleagues[22] while other parts of vmPFC were labelled using the fourth edition of *Atlas of the Human Brain*[23] (https://www.thehumanbrain.info/).

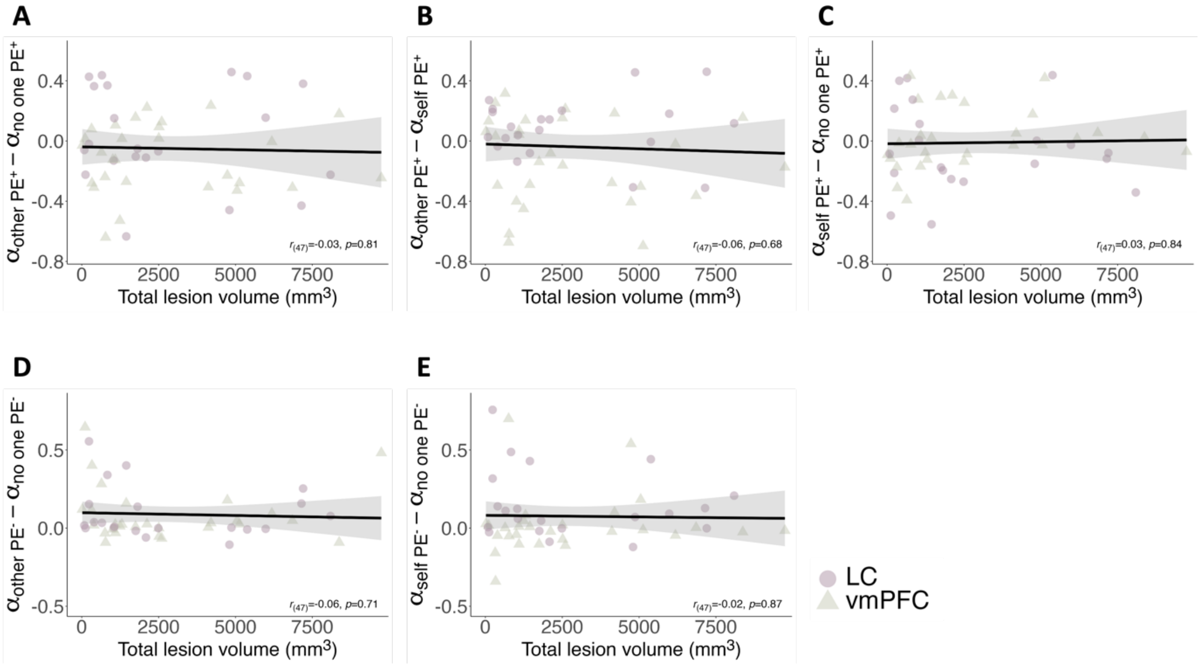

Supplementary Figure 1 Non-significant correlations between total lesion volume and VLSM predictors. To validate the voxel-based lesion symptom mapping (VLSM), we conducted a control analysis of the correlations between total lesion size and each of the five behavioural predictors. The absence of significant correlations in all instances indicates that the overall lesion size did not interfere with the VLSM analysis, confirming that the results are specifically linked to damage in the identified subregions.

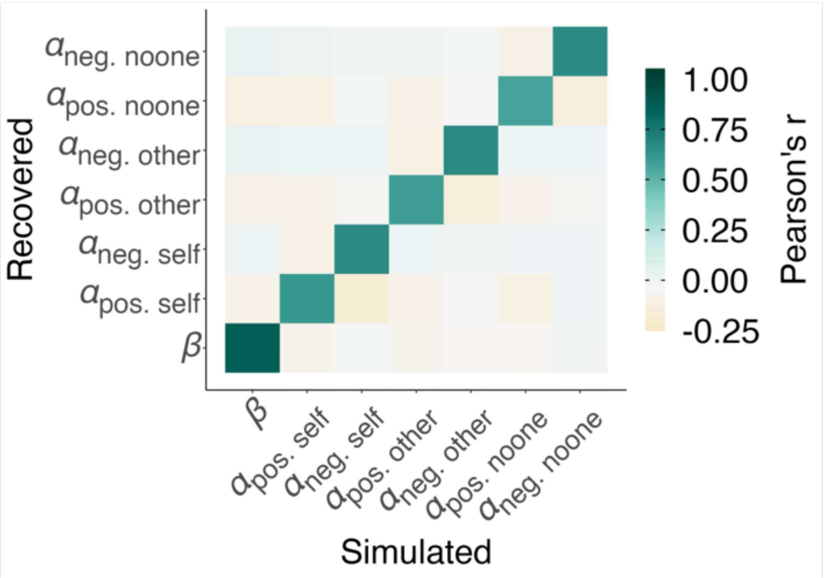

Supplementary Figure 2 Parameter recovery. Strong parameter recovery for the winning model is shown by the high correlations between simulated and recovered parameters on the diagonal and low correlations off the diagonal. Simulations for parameter recovery used a grid of values for α (0.2, 0.5, 0.8) and β (0.01, 0.11, 0.21, 0.31, 0.41, 0.51), with added noise drawn from a normal distribution * 0.5.

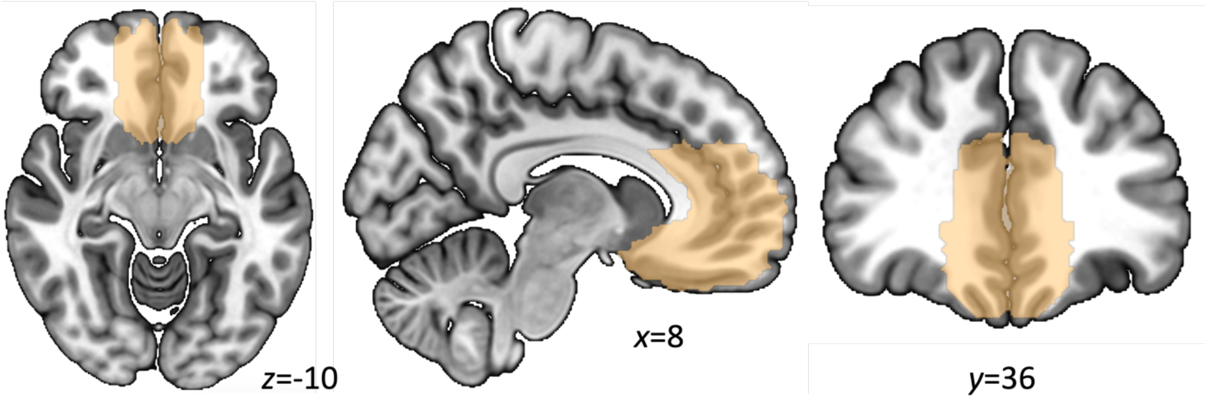

Supplementary Figure 3 Voxels included in voxel-based lesion-symptom mapping (VLSM) analysis including at least five patients with damage. We conducted VLSM in all patients with damage *n*=49), including only voxels where at least five patients had damage (shown in copper; see Methods).

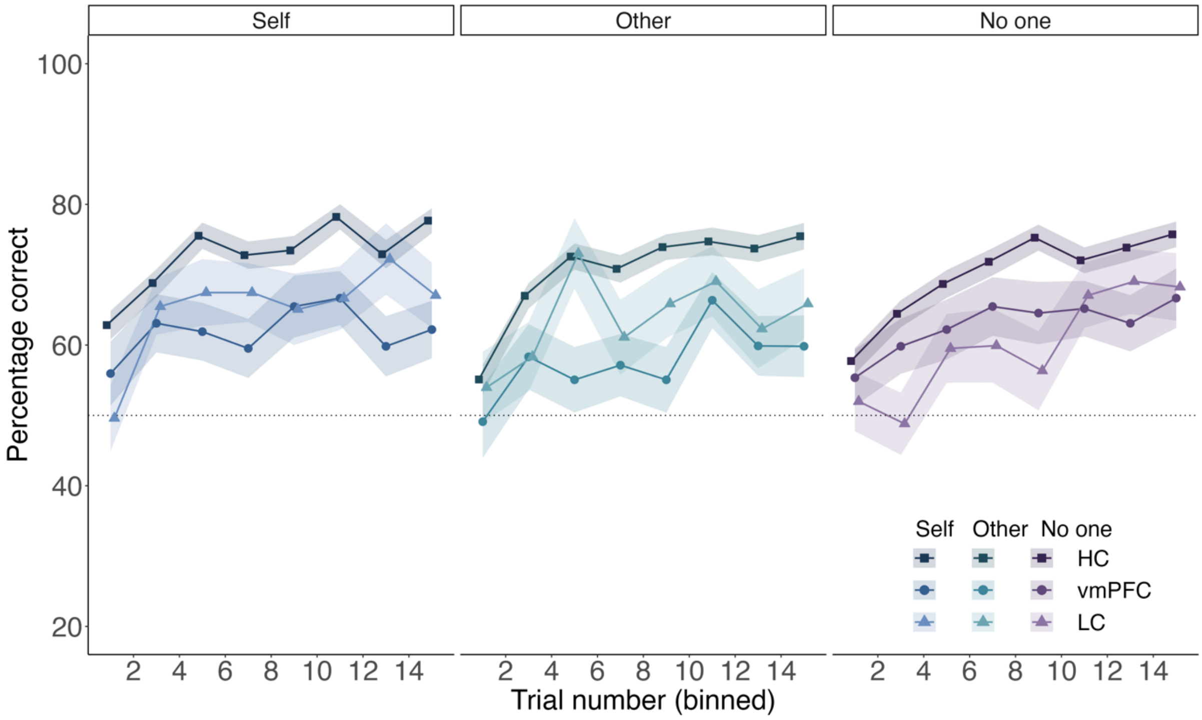

Supplementary Figure 4 Time course of accuracy across pairs of trials for each group and recipient. Learning curves showing choices in the three recipient conditions (self: blue, other: green, no one: purple) for each group (healthy controls: squares, patients with vmPFC lesion: circles, lesion controls: triangles). Accuracy on each trial number was averaged over the three blocks for the self, other and no one recipients (three blocks of 16 trials were presented for each recipient), and was then binned across pairs of trials for visualisation purposes. Points show group mean and error bars are standard error of the mean. HC: healthy controls, vmPFC: patients with ventromedial prefrontal cortex damage, LC: lesion controls.

Supplementary Table 1 Summary of demographic variables across the three groups

**Variable**	**HC** mean [SD]	**vmPFC** mean [SD]	**LC** mean [SD]	**HC vs. vmPFC** *p*-value	**LC vs. vmPFC** *p*-value	**HC vs. LC** *p*-value
Age	52.94 [22]	57.43 [11]	56.33 [10]	0.84	0.41	0.82
AMI	1.17 [0.45]	1.38 [0.57]	1.45 [0.66]	0.14	0.47	0.04
BDI	6.29 [7.73]	10.37 [7.97]	12.67 [10.25]	0.003	0.50	0.001
AE	32.65 [5.69]	31.19 [6.54]	34.00 [4.83]	0.43	0.14	0.23
CE	58.48 [7.7]	56.48 [9.52]	57.48 [7.27]	0.62	0.90	0.66
Trail A	27.11 [12.31]	26.63 [10.05]	30.17 [13.45]	0.99	0.40	0.33
Trail B	58.79 [28.95]	72.14 [44.53]	82.91 [55.85]	0.28	0.40	0.023

Note. HC: Healthy controls, LC: Lesion controls, SD: standard deviation of the mean, AMI: Apathy Motivation Index, BDI: Beck Depression Inventory, AE: Affective Empathy subscale of the Questionnaire of Cognitive and Affective Empathy, CE: Cognitive Empathy subscale of the Questionnaire of Cognitive and Affective Empathy, Trail A / B: Trail Making Test Part A / Part B, M: male; *p*-values for comparisons between groups are from Wilcoxon two-sided signed rank tests.

Supplementary Table 2 Generalised linear mixed effects model predicting accuracy

	**OR**	**95% CI**		***Z***	***p***
		**Lower**	**Upper**		
**Intercept**	1.44	1.00	2.09	1.95	0.051
**Trial number**	1.18	1.05	1.33	2.73	**0.006****
**Recipient**					
Other *vs*. Self	1.23	1.04	1.45	2.41	**0.016***
Other *vs*. No one	1.28	1.08	1.52	2.89	**0.004****
**Group**					
vmPFC *vs*. HC	2.06	1.37	3.10	3.45	**<0.001*****
vmPFC *vs*. LC	1.32	0.75	2.32	0.96	.34
**Trial number * Recipient**					
Other *vs*. Self	0.90	0.76	1.06	-1.23	0.22
Other *vs*. No one	0.98	0.83	1.16	-0.26	0.80
**Trial number * Group**					
vmPFC *vs*. HC	1.14	1.00	1.30	1.94	0.052
vmPFC *vs*. LC	0.96	0.80	1.15	-0.47	0.64
**Recipient * Group**					
vmPFC *vs*. HC: Other *vs*. Self	0.92	0.76	1.11	-0.89	0.38
vmPFC *vs*. HC: Other *vs*. No one	0.76	0.63	0.91	-2.91	**0.004****
vmPFC *vs*. LC: Other *vs*. Self	0.88	0.68	1.14	-0.94	0.35
vmPFC *vs*. LC: Other *vs*. No one	0.67	0.52	0.86	-3.13	**0.002****
**Trial number * Recipient * Group**					
vmPFC *vs*. HC: Other *vs*. Self	1.03	0.86	1.25	0.34	0.73
vmPFC *vs*. HC: Other *vs*. No one	1.02	0.84	1.23	0.21	0.83
vmPFC *vs*. LC: Other *vs*. Self	1.18	0.92	1.53	1.28	0.20
vmPFC *vs*. LC: Other *vs*. No one	1.21	0.93	1.56	1.44	0.15

Note. HC: healthy controls, vmPFC: patients with ventromedial prefrontal cortex damage, LC: lesion control with brain damage elsewhere, OR: odds ratio, 95% CI: 95% confidence interval, *Z*: *Z*-value of effect, *p*: *p*-value of effect. Bolded *p*-values are statistically significant, and asterisks represent the level significance (*p<0.05, **p<0.01, ***p<0.001).

Supplementary Table 3 Post-hoc tests for accuracy

	Contrast	OR	SE	*Z*-ratio	FDR-corrected *p* value
**Group: HC**	Other *vs.* Self	0.89	0.04	-2.70	**0.025***
	Other *vs.* No one	1.03	0.05	0.74	0.55
	Self *vs.* No one	1.17	0.05	3.44	**0.005****
**Group: vmPFC**	Other *vs.* Self	0.81	0.07	-2.41	**0.035***
	Other *vs.* No one	0.78	0.07	-2.89	**0.017***
	Self *vs.* No one	0.96	0.08	-0.50	0.65
**Group: LC**	Other *vs.* Self	0.92	0.09	-0.84	0.52
	Other *vs.* No one	1.17	0.11	1.63	0.15
	Self *vs.* No one	1.27	0.13	2.46	**0.035***
**HC *vs.* vmPFC**	Self	0.53	0.11	-3.04	**0.014***
	Other	0.49	0.10	-3.45	**0.005****
	No one	0.64	0.13	-2.11	0.070
**HC *vs.* LC**	Self	1.62	0.38	2.04	0.074
	Other	1.56	0.37	1.89	0.096
	No one	1.77	0.42	2.43	**0.035***
**LC *vs.* vmPFC**	Self	0.86	0.25	-0.54	0.65
	Other	0.76	0.22	-0.96	0.47
	No one	1.14	0.33	0.46	0.65

Notes. HC: healthy controls, vmPFC: patients with ventromedial prefrontal cortex damage, LC: lesion control with brain damage elsewhere, OR: odds ratio, SE: standard error, FDR: false discovery rate. Bolded *p*-values are statistically significant, and asterisks represent the level of significance (*p<0.05, **p<0.01, ***p<0.001).

Supplementary Table 4 Model selection criteria across the full model space

**Model**	**ΔBIC_{int} (Lower = better)**			**LME (Higher = better)**		
	HC	vmPFC	LC	HC	vmPFC	LC
$1\alpha 1\beta$	879.20	139.22	202.52	-9153.36	-2385.96	-1785.99
$3\alpha 1\beta$	685.22	165.53	121.48	-9043.25	-2394.84	-1741.43
$2\alpha_{S/O+N} 1\beta$	776.90	148.42	186.56	-9090.42	-2389.43	-1772.83
$2\alpha_{O/S+N} 1\beta$	834.88	148.00	178.90	-9118.59	-2386.63	-1771.15
$2\alpha_{N/S+O} 1\beta$	751.01	170.38	114.72	-9079.56	-2397.70	-1735.66
$3\alpha 3\beta$	1012.42	231.26	165.92	-9219.11	-2419.23	-1753.70
$0\alpha 1\beta$	5841.63	483.23	618.40	-11627.54	-2557.11	-1992.68
$0\alpha 3\beta$	6033.65	549.29	656.02	-11714.31	-2580.02	-2002.87
$\boldsymbol{6\alpha_{PE} 1\beta}$	**0**	**0**	**0**	**-8687.12**	**-2298.48**	**-1672.62**
$6\alpha_{PE} 3\beta$	395.03	96.17	55.94	-8911.60	-2343.57	-1688.16
$4\alpha_{chosen} 1\beta$	648.08	96.78	138.67	-9025.81	-2352.89	-1741.55
$5\alpha_{chosen} 1\beta$	700.32	120.03	151.99	-9048.83	-2358.25	-1743.34
$6\alpha_{chosen} 1\beta$	1051.73	225.24	190.697151	-9198.49	-2394.71	-1752.07

Notes. Values are colour-coded from green (best fitting model) to red (worst fitting model). The α and β parameters represent the learning rate and temperature (i.e., decision noise) parameters, respectively. The S, O, N, PE, and chosen subscripts represent the self condition, other condition, no one condition, prediction-error-based learning rates, and chosen/unchosen-based learning rates, respectively. Bolded values denote the winning model. ΔBIC_{int}: integrated Bayesian Integrated Criterion (BIC) relative to the winning model (lower is better; 0 indicates winning model), LME: log model evidence (higher is better), HC: healthy controls, vmPFC: participants with ventromedial prefrontal cortex lesions, LC: lesion controls.

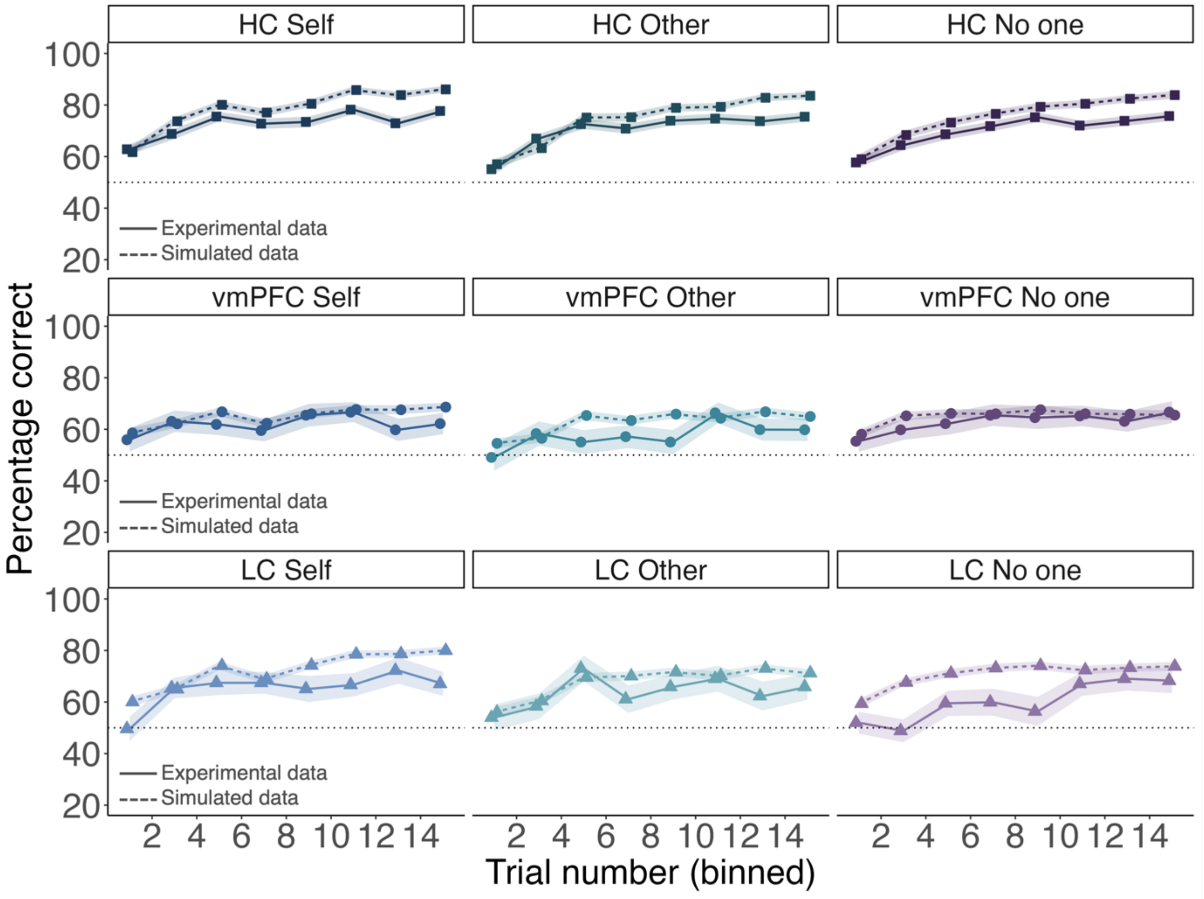

Supplementary Figure 5 Winning model captured the pattern of learning across groups and conditions. Learning curves showing real experimental data (solid lines) and simulated choices (dashed) in the three recipient conditions (self: blue, other: green, no one: purple) for each group (healthy controls: squares, patients with vmPFC lesion: circles, lesion controls: triangles). Simulated data was created using the median parameter values for each recipient for each group ($\alpha_{self\ positive\ PE}$, $\alpha_{self\ negative\ PE}$, $\alpha_{other\ positive\ PE}$, $\alpha_{other\ negative\ PE}$, $\alpha_{no\ one\ positive\ PE}$, $\alpha_{no\ one\ negative\ PE}$, β). A grid of parameter values was based on the parameter medians +/- 0.05, with noise = 0.1 was used to simulate choices (*n*=128). Accuracy on each trial number was averaged over the three blocks for the self, other and no one recipients (three blocks of 16 trials were presented for each recipient), and was then binned across pairs of trials for visualisation purposes. Points show group mean and error bars are standard error of the mean. HC: healthy controls, vmPFC: patients with ventromedial prefrontal cortex damage, LC: lesion controls.

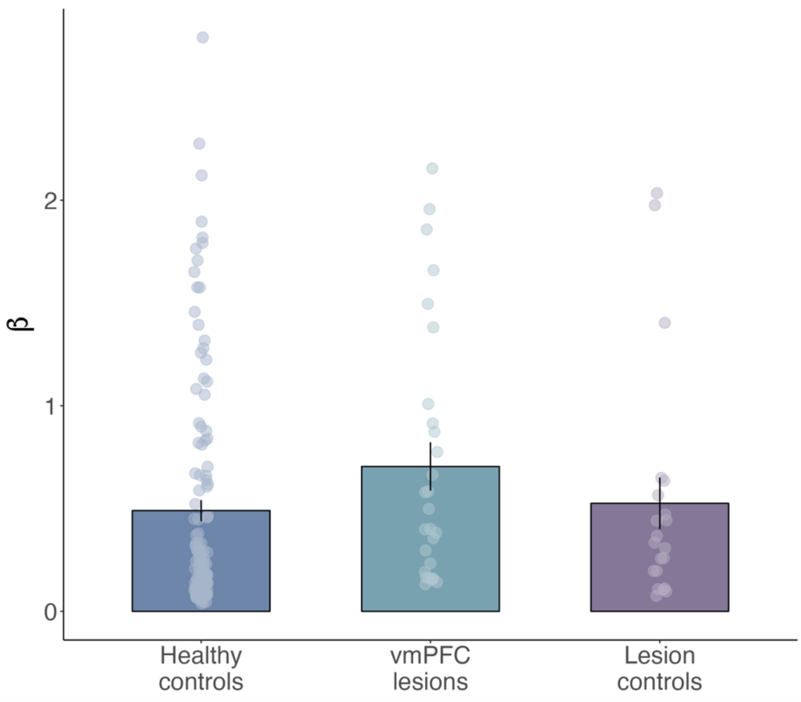

Supplementary Figure 6 Decision noise did not significantly differ between the groups. Error bars are standard error of the mean. *n*=173 (124 healthy controls, 28 patients with ventromedial prefrontal cortex damage, 21 with damage elsewhere). HC: healthy controls, vmPFC: patients with ventromedial prefrontal cortex damage, LC: lesion controls.

Supplementary Table 5 Linear mixed effects model predicting positive PE learning rates

	OR	95% CI		Z	*p*
		Lower	Upper		
**Intercept**	1.66	1.58	1.75	19.30	**<0.001*****
**Recipient**					
Other *vs.* Self	1.14	1.06	1.22	3.59	**<0.001*****
Other *vs.* No one	1.12	1.04	1.20	3.06	**0.002****
**Group**					
vmPFC *vs.* HC	0.90	0.85	0.96	-3.53	**<0.001*****
vmPFC *vs.* LC	1.03	0.95	1.11	0.67	0.50
**Recipient * Group**					
vmPFC *vs.* HC: Other *vs.* Self	0.97	0.90	1.05	-0.68	0.49
vmPFC *vs.* HC: Other *vs.* No one	0.84	0.78	0.91	-4.36	**<0.001*****
vmPFC *vs.* LC: Other *vs.* Self	0.81	0.73	0.90	-3.87	**<0.001*****
vmPFC *vs.* LC: Other *vs.* No one	0.87	0.78	0.97	-2.58	**0.010***

Notes. OR: odds ratio, 95% CI: 95% confidence interval, *Z*: *Z*-value of effect, *p*: *p*-value of effect. Bolded *p*-values are statistically significant, and asterisks represent the level significance (*p<0.05, **p<0.01, ***p<0.001).

Supplementary Table 6 Post-hoc tests for positive PE learning rates

	Contrast	OR	SE	*t*-ratio	FDR-corrected *p* value
**Group: HC**	Other *vs*. Self	-0.10	0.02	-5.96	**<0.001*** **
	Other *vs*. No one	0.06	0.02	3.71	**<0.001*** **
	Self *vs*. No one	0.16	0.02	9.67	**<0.001*** **
**Group: vmPFC**	Other *vs*. Self	-0.13	0.04	-3.59	**<0.001*** **
	Other *vs*. No one	-0.11	0.04	-3.06	**0.004** **
	Self *vs*. No one	0.02	0.04	0.53	0.60
**Group: LC**	Other *vs*. Self	0.08	0.04	2.01	0.062
	Other *vs*. No one	0.03	0.04	0.76	0.50
	Self *vs*. No one	-0.05	0.04	-1.25	0.25
**HC *vs*. vmPFC**	Self	0.13	0.03	4.45	**<0.001*** **
	Other	0.10	0.03	3.53	**<0.001*** **
	No one	0.28	0.03	9.45	**<0.001*** **
**HC *vs*. LC**	Self	0.05	0.03	1.66	0.12
	Other	-0.13	0.03	-3.94	**<0.001*** **
	No one	-0.16	0.03	-4.91	**<0.001*** **
**LC *vs*. vmPFC**	Self	0.19	0.04	4.59	**<0.001*** **
	Other	-0.03	0.04	-0.67	0.53
	No one	0.11	0.04	2.83	**0.007** **

Notes. HC: healthy controls, vmPFC: patients with ventromedial prefrontal cortex damage, LC: lesion control with brain damage elsewhere, OR: odds ratio, SE: standard error, Z: Z-value of effect, FDR: false discovery rate. Bolded *p*-values are statistically significant, and asterisks represent the level of significance (**p*<0.05, ***p*<0.01, ****p*<0.001).

Supplementary Table 7 Linear mixed effects model predicting negative PE learning rates

	OR	95% CI		Z	*p*
		Lower	Upper		
**Intercept**	1.24	1.18	1.30	8.57	**<0.001*** **
**Recipient**					
Other *vs*. Self	0.94	0.89	0.98	-2.58	**0.010***
Other *vs*. No one	0.92	0.87	0.97	-3.38	**<0.001*** **
**Group**					
vmPFC *vs*. HC	0.93	0.88	0.98	-2.54	**0.011***
vmPFC *vs*. LC	0.99	0.92	1.07	-0.24	0.81
**Recipient * Group**					
vmPFC *vs*. HC: Other *vs*. Self	1.06	1.00	1.12	2.11	**0.035***
vmPFC *vs*. HC: Other *vs*. No one	1.03	0.97	1.08	0.95	0.34
vmPFC *vs*. LC: Other *vs*. Self	1.13	1.05	1.22	3.16	**0.002****
vmPFC *vs*. LC: Other *vs*. No one	0.99	0.92	1.07	-0.25	0.80

Notes. OR: odds ratio, 95% CI: 95% confidence interval, *Z*: *Z*-value of effect, *p*: *p*-value of effect. Bolded *p*-values are statistically significant, and asterisks represent the level significance (*p<0.05, **p<0.01, ***p<0.001).

Supplementary Table 8 Post-hoc tests for negative PE learning rates

	**Contrast**	**OR**	**SE**	***t*-ratio**	**FDR-corrected *p* value**
**Group: HC**	Other *vs.* Self	0.01	0.01	0.50	0.70
	Other *vs.* No one	0.06	0.01	4.91	**<0.001*** **
	Self *vs.* No one	0.05	0.01	4.91	**<0.001*** **
**Group: vmPFC**	Other *vs.* Self	0.06	0.03	2.58	**0.023***
	Other *vs.* No one	0.08	0.03	3.38	**0.003****
	Self *vs.* No one	0.02	0.03	0.81	0.55
**Group: LC**	Other *vs.* Self	-0.06	0.03	-1.95	0.085
	Other *vs.* No one	0.09	0.03	3.26	**0.004****
	Self *vs.* No one	0.15	0.03	5.20	**<0.001*** **
**HC *vs.* vmPFC**	Self	0.01	0.03	0.40	0.73
	Other	0.07	0.03	2.54	**0.023***
	No one	0.04	0.03	1.58	0.17
**HC *vs.* LC**	Self	-0.12	0.03	-3.98	**<0.001*** **
	Other	-0.06	0.03	-1.96	0.085
	No one	-0.02	0.03	-0.80	0.55
**LC *vs.* vmPFC**	Self	-0.11	0.04	-2.96	**0.008****
	Other	0.01	0.04	0.24	0.81
	No one	0.02	0.04	0.49	0.70

Notes. HC: healthy controls, vmPFC: patients with ventromedial prefrontal cortex damage, OR: odds ratio, SE: standard error, Z: Z-value of effect, FDR: false discovery rate. Bolded *p*-values are statistically significant, and asterisks represent the level of significance (**p*<0.05, ***p*<0.01, ****p*<0.001).

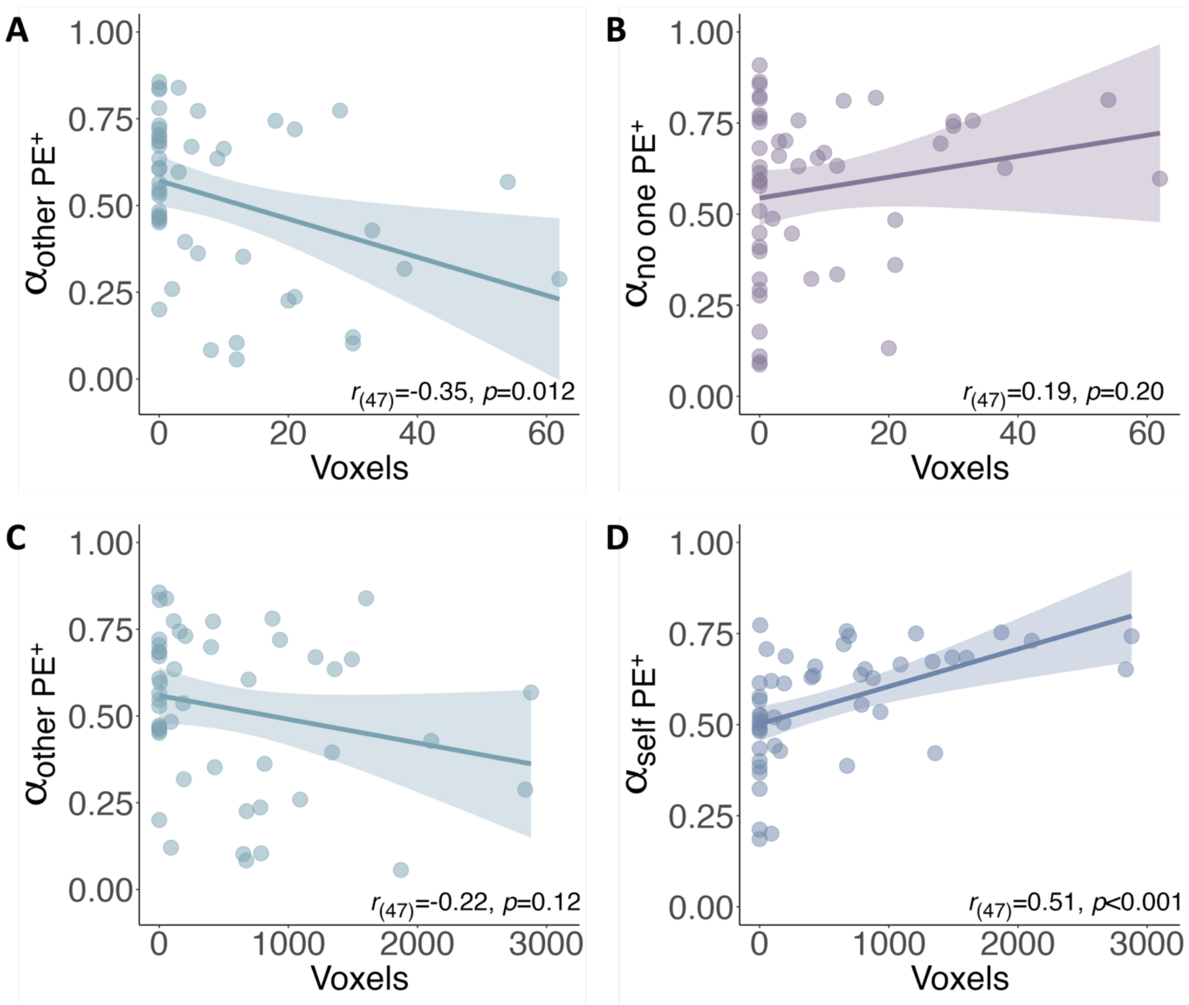

Supplementary Figure 7 Correlations between positive PE learning rate parameters for each recipient and damage in regions identified in VLSM analysis. As a follow-up to the voxel-based lesion-symptom mapping (VLSM) analysis, we plotted damage in the identified areas against learning rates for positive PE for each recipient separately. Note that these graphs are provided for visualization and interpretation of the VLSM results and are not intended for quantitative analysis. Top row: first, we plotted damage in the sgACC area found to be negatively associated with the other-no one difference in learning rates against positive PE learning rates for **(A)** other and **(B)** no one. Bottom row: next, we plotted damage in the broader vmPFC area found to be negatively associated with the other-self difference against positive PE learning rates for **(C)** other and **(D)** self.

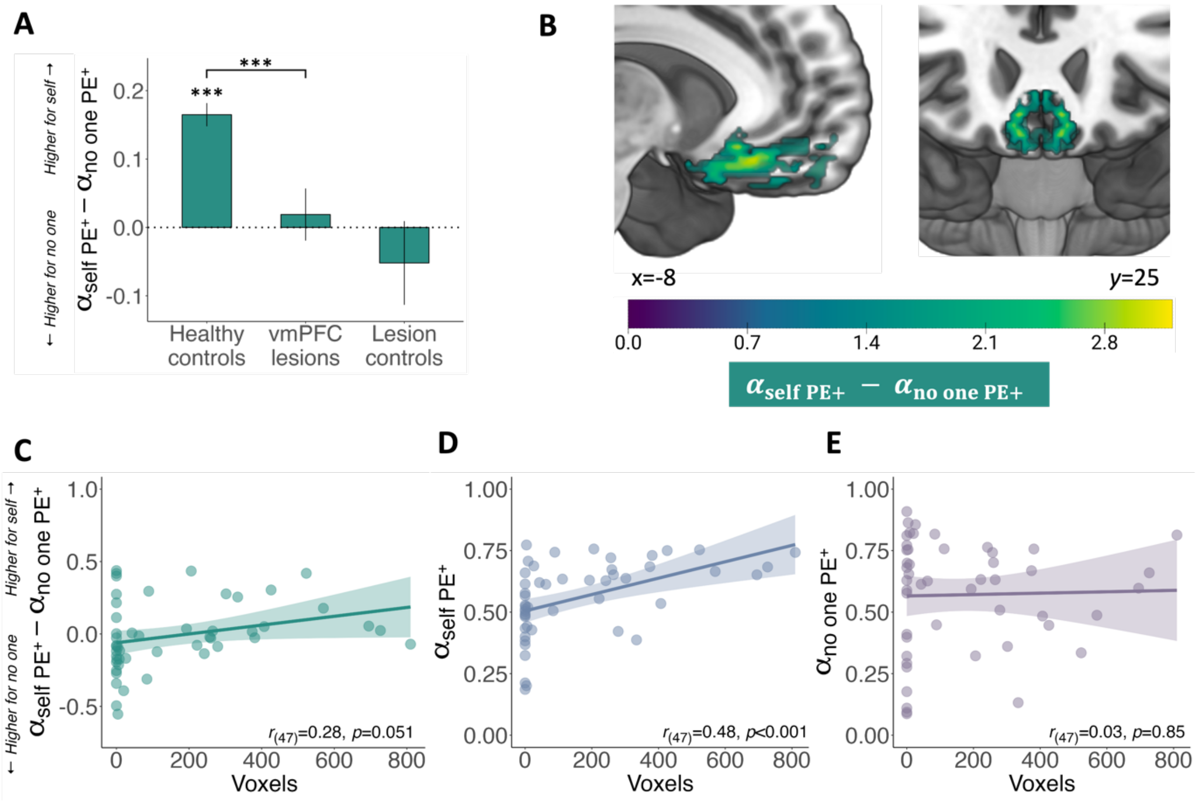

Supplementary Figure 8 Damage to lateral portions of vmPFC was positively associated with the difference between self and no one in learning rates for positive PE. (A) Healthy controls had higher learning rates for positive PEs for self relative to no one, a pattern absent after vmPFC damage. **(B)** Permutation-based, whole-brain, non-parametric voxel-based lesion mapping (VLSM) reveals that damage to lateral parts of vmPFC (area 13; peak 8, 26, -12) is associated with the difference between self and no one learning rates for positive PEs. **(C)** Plotting the association between the difference in positive alphas for self-no one against the extent of damage in the area identified in the relevant VLSM analysis. As a follow-up, we plotted each recipient separately to examine how damage in the identified area affected learning rates for positive PEs **(D)** for self and **(E)** for no one. Note that graphs **(C)**, **(D)**, and **(E)** are provided for visualization and interpretation of the VLSM results and are not intended for quantitative analysis. N = 49 (28 patients with ventromedial prefrontal cortex damage, 21 with damage elsewhere). PE: prediction error, HC: healthy controls, vmPFC: patients with ventromedial prefrontal cortex damage, LC: lesion controls. *p<0.05, **p<0.01, ***p<0.001.

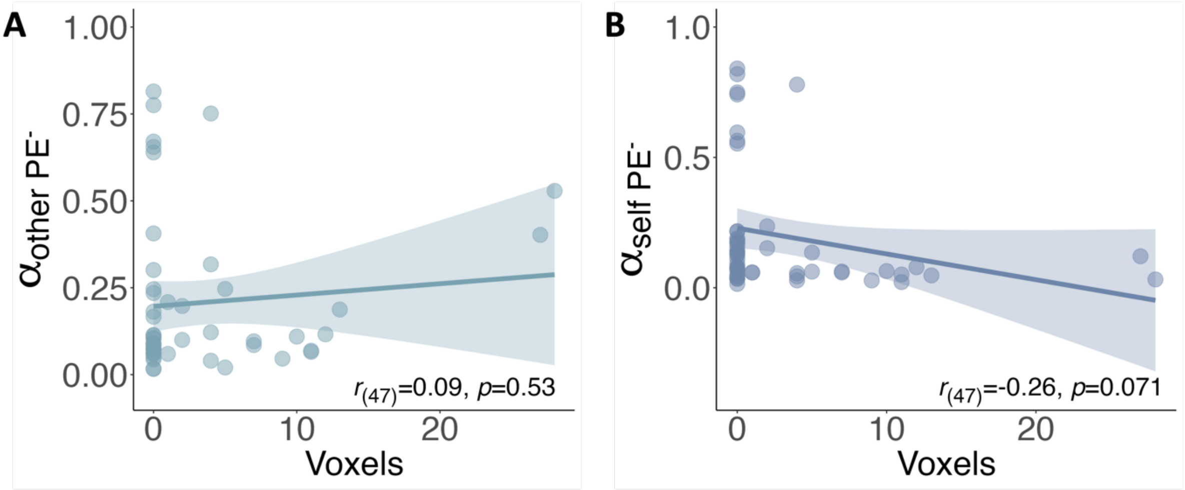

Supplementary Figure 9 Correlations of individual negative PE parameters for other and self with damage in identified vmPFC regions. As a follow-up to the voxel-based lesion-symptom mapping (VLSM) analysis, we plotted each recipient separately to examine how damage in the identified regions (sgACC and ACCg) affected learning rates for negative PEs **(A)** for other and **(B)** for self. Note that these graphs are provided for visualization and interpretation of the VLSM results and are not intended for quantitative analysis.

Supplementary Table 9 Results of VLSM analyses with peak coordinates

	Voxels	MNI coordinates			*p*
		x	y	z	
**Learning rates for positive PEs**					
Other – No one	33	±4	20	-4	**0.005****
Other – Self	3452	8	32	-20	**<0.001*****
Self – No one	847	8	26	-12	**0.007****
**Learning rates for negative PEs**					
Other – Self	3	8	6	-16	**0.013***
	1	±4	10	-10	**0.016***
	1	±2	34	8	**0.017***
Self – No one	99	±8	14	-22	**0.004****

Notes. PE: prediction error, MNI: Montreal Neurological Institute. Bolded *p*-values are statistically significant, and asterisks represent the level of significance (*p<0.05, **p<0.01, ***p<0.001).

References

1. Ang YS, Lockwood P, Apps MA, Muhammed K, Husain M. Distinct subtypes of apathy revealed by the apathy motivation index. *PloS One*. 2017;12(1):e0169938.

2. Beck AT, Steer RA, Brown GK. Beck depression inventory. Published online 1996.

3. Reniers RL, Corcoran R, Drake R, Shryane NM, Völlm BA. The QCAE: A questionnaire of cognitive and affective empathy. *J Pers Assess*. 2011;93(1):84-95.

4. Reitan RM. The relation of the trail making test to organic brain damage. *J Consult Psychol*. 1955;19(5):393.

5. Lüdecke D, Ben-Shachar MS, Patil I, Makowski D. Extracting, computing and exploring the parameters of statistical models using R. *J Open Source Softw*. 2020;5(53):2445.

6. Cutler J, Wittmann MK, Abdurahman A, et al. Ageing is associated with disrupted reinforcement learning whilst learning to help others is preserved. *Nat Commun*. 2021;12(1):1-13.

7. Scholl J, Kolling N, Nelissen N, Wittmann MK, Harmer CJ, Rushworth MF. The good, the bad, and the irrelevant: neural mechanisms of learning real and hypothetical rewards and effort. *J Neurosci*. 2015;35(32):11233-11251.

8. Palminteri S, Lefebvre G, Kilford EJ, Blakemore SJ. Confirmation bias in human reinforcement learning: Evidence from counterfactual feedback processing. *PLoS Comput Biol*. 2017;13(8):e1005684.

9. Matlab. *9.7.0.1190202 (R2019b)*. The Mathworks Inc.; 2018.

10. Wittmann MK, Fouragnan E, Folloni D, et al. Global reward state affects learning and activity in raphe nucleus and anterior insula in monkeys. *Nat Commun*. 2020;11(1):3771.

11. Huys QJ, Cools R, Gölzer M, et al. Disentangling the roles of approach, activation and valence in instrumental and pavlovian responding. *PLoS Comput Biol*. 2011;7(4):e1002028.

12. Daw ND. Trial-by-trial data analysis using computational models. *Decis Mak Affect Learn Atten Perform XXIII*. 2011;23(1):3-38.

13. MacKay DJ. *Information Theory, Inference and Learning Algorithms*. Cambridge university press; 2003.

14. Stephan KE, Penny WD, Daunizeau J, Moran RJ, Friston KJ. Bayesian model selection for group studies. *Neuroimage*. 2009;46(4):1004-1017.

15. Penny WD, Friston KJ, Ashburner JT, Kiebel SJ, Nichols TE. *Statistical Parametric Mapping: The Analysis of Functional Brain Images*. Elsevier; 2011.

16. Lockwood PL, Apps MA, Valton V, Viding E, Roiser JP. Neurocomputational mechanisms of prosocial learning and links to empathy. *Proc Natl Acad Sci*. 2016;113(35):9763-9768.

17. Smith SM, Jenkinson M, Woolrich MW, et al. Advances in functional and structural MR image analysis and implementation as FSL. *Neuroimage*. 2004;23:S208-S219.

18. Smith SM, Nichols TE. Threshold-free cluster enhancement: Addressing problems of smoothing, threshold dependence and localisation in cluster inference. *NeuroImage*. 2009;44(1):83-98. doi:10.1016/j.neuroimage.2008.03.061

19. Winkler AM, Ridgway GR, Webster MA, Smith SM, Nichols TE. Permutation inference for the general linear model. *NeuroImage*. 2014;92:381-397. doi:10.1016/j.neuroimage.2014.01.060

20. Su Z, Garvert M, Zhang L, et al. Dorsomedial and ventromedial prefrontal cortex lesions differentially impact social influence and temporal impulsivity.

21. Lockwood PL, Cutler J, Drew D, et al. Human ventromedial prefrontal cortex is necessary for prosocial motivation. *Nat Hum Behav*. Published online 2024:1-14.

22. Palomero-Gallagher N, Eickhoff SB, Hoffstaedter F, et al. Functional organization of human subgenual cortical areas: relationship between architectonical segregation and connectional heterogeneity. *Neuroimage*. 2015;115:177-190.

23. Mai JK, Majtanik M, Paxinos G. *Atlas of the Human Brain*. Academic Press; 2015.
